# TAF15 mediates ROP16-induced apoptosis and cell cycle arrest in lung cancer

**DOI:** 10.1186/s13071-025-06933-6

**Published:** 2025-07-19

**Authors:** Guangqi Li, Mei Tian, Yuning Zhou, Shaohan Ma, Shanni Ma, Qirui Ge, Zhijun Zhao

**Affiliations:** 1 Medical Laboratory Center, General Hospital of Ningxia Medical University, Yinchuan, Ningxia China; 2 Ningxia Key Laboratory of Clinical Pathogenic Microorganisms, Yinchuan, Ningxia China; 3 School of Laboratory Technology, Ningxia Medical University, Yinchuan, Ningxia China; 4 School of Clinical Medicine, Ningxia Medical University, Yinchuan, Ningxia China; 5 Central Laboratory, Peking University First Hospital Ningxia Women and Children’s Hospital (Ningxia Hui Autonomous Region Maternal and Child Health Hospital), Yinchuan, Ningxia China

**Keywords:** Lung adenocarcinoma, ROP16, Toxoplasma gondii, TAF15, Cell cycle, Apoptosis

## Abstract

**Objective:**

This study aimed to investigate the effects of the interaction between ROP16 and TAF15 on apoptosis and cell cycle regulation in A549 lung adenocarcinoma cells.

**Methods:**

Lentivirus-infected A549 cells which could overexpress type I, II, and III ROP16, along with an empty vector control group, were established. Potential interacting proteins with ROP16 were identified by using co-immunoprecipitation (Co-IP) and liquid chromatography-mass spectrometry (LC–MS). High-scoring interacting proteins were selected for verification through Venn diagram analysis, scoring, and intensity evaluation. The interaction between ROP16 and TAF15 was confirmed by using co-immunoprecipitation. Moreover, TAF15-specific siRNA was synthesized and transfected into A549 cells overexpressing ROP16 types I, II, and III. The levels of apoptosis and cell cycle were detected by flow cytometry (FCM), and the expression levels of apoptosis-related proteins and cell cycle-related proteins were detected by real-time fluorescence quantitative PCR (RT-qPCR) and western blotting.

**Results:**

Three stable A549 cell lines overexpressing type I, II, and III ROP16 were successfully established. IP-LC/MS identified 29 potential ROP16-interacting proteins, among which 13 had a score greater than 100. Subsequent bioinformatic analysis and co-immunoprecipitation confirmed the interaction between ROP16 and TAF15. Flow cytometry analysis revealed that type I and III ROP16 promoted A549 cell apoptosis and induced cell cycle arrest. Furthermore, western blotting and RT-qPCR demonstrated their modulation of the expression of cell cycle regulators (p21, CDK6, Cyclin D1) and apoptosis-related proteins (Bax, BCL-2, p53, Caspase-9). In contrast, type II ROP16 exhibited none of these effects. However, upon TAF15 silencing, the pro-apoptotic effects of type I and III ROP16 were attenuated, no significant cell cycle arrest was observed, and their regulatory effects on the expression of cell cycle- and apoptosis-related proteins were also significantly diminished.

**Conclusions:**

Our study demonstrates that type I/III ROP16 induce apoptosis and cell cycle arrest in A549 cells through interactions with the host RNA-binding protein TAF15. These findings not only provide compelling evidence for the oncosuppressive potential of *Toxoplasma gondii*-derived secretory proteins but also uncover a previously unrecognized mechanism by which parasite effectors hijack host transcriptional regulators to subvert cancer cell survival pathways.

**Graphical Abstract:**

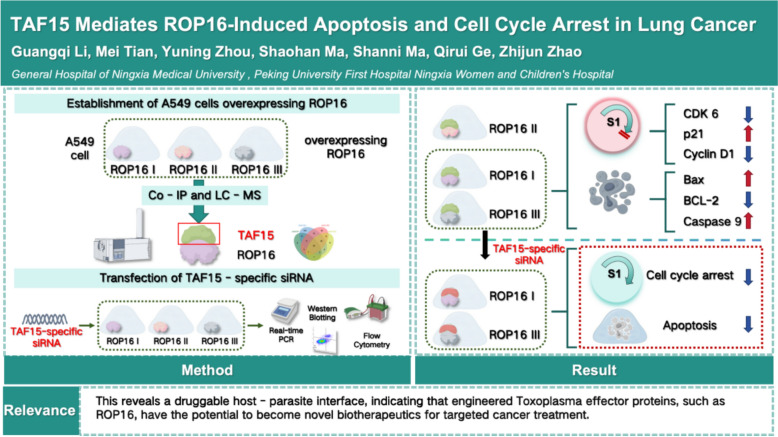

**Supplementary Information:**

The online version contains supplementary material available at 10.1186/s13071-025-06933-6.

## Background

Emerging research has revealed a significant correlation between various parasitic infections and the genesis and progression of cancer, positioning the anti-tumor effector of parasites as a burgeoning area of interest within oncological therapeutics [1]. Specifically, parasites such as *Trichuris trichiura*, *Ascaris lumbricoides*, and *Toxoplasma gondii* have demonstrated the capacity to suppress tumor cell proliferation in malignancies including breast, colorectal, and lung cancers [1–3]. *Toxoplasma gondii*, an opportunistic protozoan, is estimated to infect roughly 30% of the global population, over 90%, of which exhibiting no clinical symptoms [4]. Investigations have indicated that the presence of *Toxoplasma gondii* within the host or tumor microenvironment can mitigate the immunosuppressive state of myeloid cells, thereby conferring anti-tumor efficacy [5]. In addition, the effector molecules secreted by *Toxoplasma gondii*, such as rhoptry proteins (ROPs) and dense granule antigens (GRAs), can stimulate the host immune system to suppress tumors. Moreover, these proteins themselves can participate in the regulation of intracellular signaling pathways in tumor cells and the transcription and translation of certain proteins, exhibiting strong antitumor effects [6].

Lung cancer, a common malignant neoplasm originating from the bronchial mucosa or pulmonary glands, has the highest global incidence and mortality rates [7, 8]; it is classified into small cell lung cancer (SCLC) and non-small cell lung cancer (NSCLC), with NSCLC accounting for the majority of cases, including lung adenocarcinoma, which constitutes approximately 40% of all lung cancer cases [9]. Lung adenocarcinoma patients exhibit a high mortality rate, with a 5-year survival rate of only 23% [10]. Current treatment modalities primarily include surgical resection, traditional radiotherapy, and chemotherapy-based combination therapies [11–13]. The emergence of drug resistance and the significant heterogeneity in patient responses to these adjuvant therapies substantially limit their overall efficacy [14, 15]. Additionally, the development of new pharmacological agents has been relatively slow, resulting in a limited arsenal of effective treatment options for clinicians. However, looking ahead, biological therapies, including the development of novel biologics and immunotherapies, present a promising frontier in the management of lung adenocarcinoma. These novel strategies aim to leverage the body’s immune system and the molecular characteristics of tumors to enhance treatment efficacy and improve patient outcomes. Continued research and clinical trials are essential to explore the full potential of these emerging therapies, which may ultimately transform the therapeutic landscape for lung adenocarcinoma patients.

Despite the strong anti-tumor effects demonstrated by the parasite, tissue damage caused by the parasite itself is unavoidable. However, utilizing proteins secreted by the parasite with unique functions can mitigate this issue. Rhoptries are the critical organelles within *Toxoplasma gondii* that play essential roles in parasite invasion, establishment of replicative niches in parasitic vesicles, manipulation of host cells, and resistance to innate immunity stimulated by host IFN-γ [16]. Additionally, rhoptry protein 16 (ROP16), a key protein secreted by rhoptries, regulates host-cell signaling and is a significant virulence factor [17]. *Toxoplasma gondii* is classified into three types based on virulence factors: I/II/III. The amino acid sequences of ROP16 secreted by different *Toxoplasma gondii* genotypes are polymorphic, and some ROP16 possess serine/threonine kinase activity [18]. These proteins can rapidly penetrate the nuclear membrane and enter the nucleus within 10 min of invasion, and regulating host cell signaling pathways, significantly affecting cellular activities [19, 20]. A number of studies have shown that when overexpressed in cells ROP16 is able to affect cell proliferation and apoptosis [21–23]. However, the mechanism by which ROP16 interacts with specific protein partners to suppress malignant phenotypes in lung adenocarcinoma cells remains unclear.

We primarily investigated the effect of *Toxoplasma gondii* ROP16 on apoptosis and cell cycle, as well as the influence of the expression of cell cycle-related and apoptosis-related proteins to identify the signaling pathways regulated by ROP16 that can inhibit tumor proliferation. To determine whether ROP16 interacts with intracellular proteins in lung adenocarcinoma cells and if these interactions influence ROP16 anti-tumor effects, we used IP-LC/MS to identify ROP16-interacting proteins in lung adenocarcinoma cells. Through bioinformatic analysis, we are interested in that TAF15, an RNA-binding protein, dentified as a highly interacting protein with ROP16. Additionally, TAF15 is a key regulator of gene expression, involved in various kinetic functions such as RNA splicing, polyadenylation, capping, modification, export, localization, translation, and transport [24, 25]. We silenced TAF15 in A549 cells overexpressing type I, II and III ROP16 to examine its impact on ROP16-mediated anti-tumor functions, thereby exploring potential mechanisms underlying these effects.

## Methods

### Cell culture of A549 lung adenocarcinoma cells

The A549 human lung adenocarcinoma cell line was obtained from the Shanghai Cellular Research Institute, Chinese Academy of Science (Shanghai, China) and stored at the Ningxia Key Laboratory of Clinical and Pathogenic Microbiology, General Hospital of Ningxia Medical University, Yinchuan, China. Cells were routinely cultured in Dulbecco’s Modified Eagle’s Medium (DMEM) supplemented with 10% fetal bovine serum (FBS) and passaged as needed.

### Lentiviral transfection and stable overexpression cell line construction

Gene sequences of three strains of *T. gondii*, namely, RH (type I), Me49 (type II), and VEG (type III), were obtained from the website (https://toxodb.org/toxo/app), enzymatically cleaved, and ligated with pHBLV-CMV-MCS-EF1-Zsgreen1-T2A-puro. 6 × His tags were added, and lentiviral expression vectors were constructed, using pSPAX2, pMD2G, and shuttled plasmid for lentiviral packaging. The lentiviral vector construction was provided by Hanbio Biotechnology Co., Ltd, and the PCR primers were produced by Shanghai Sangong Biotechnology Co., LTD. The detailed sequence information, primer sequences, and plasmid maps involved in this study were provided in Text S1. A549 cells were transfected with the lentiviral vector overexpressing ROP16, and the cells were screened with puromycin for 2 weeks to obtain stable cell lines overexpressing ROP16. RT-qPCR, western blotting, and immunofluorescence assay were used to verify the successful transfection and expression of ROP16.

### Immunofluorescence assay

A 12-well cell culture plate was taken, and a 20 mm cell culture cover slip was placed in each well. 1 × 10^5^ cells per group were seeded and cultured for 24 h until they reached approximately 70% confluence. 1 mL of 4% paraformaldehyde was added to each well, and the cells were fixed at room temperature for 20 min. After aspirating the fixing solution, precooled PBS buffer was added to rinse the cells for 5 min; this process was repeated three times. 1 mL of 0.5% Triton X-100 was added to each well to permeabilize the cells for 10 min, followed by additional rinsing with PBS. Then, 3% BSA was added to each well and incubated at room temperature for 1 h to block non-specific binding. After aspirating the blocking solution, 500 μL of the primary antibody (diluted in 2% BSA at 1:200) was added to each well and incubated overnight at 4 °C. The primary antibody solution was discarded, and the wells were rinsed with PBS. Subsequently, the fluorescent secondary antibody was added and incubated at 37 °C in the dark for 1 h. After rinsing with PBS, the cover slips were mounted using a DAPI-containing mounting medium and observed immediately under a laser confocal microscope. All experimental data were analyzed with ImageJ software.

### Co-IP and MS

To identify proteins interacting with type I(RH), II(ME49), and III(VEG) ROP16 in A549 cells, we performed IP-LC/MS comparing cells overexpressing each ROP16 genotypes to control cells. For Co-IP, the cells were cultured until reaching 80% confluence, and cell lysates were collected to enrich the proteins bound to ROP16 using BeyoMag^™^ Anti-His Magnetic Beads. These were subjected to SDS-PAGE gel electrophoresis and Coomassie Brilliant Blue staining. The target protein bands were excised, decolorized, and dehydrated with acetonitrile containing 50 mM ammonium bicarbonate, then digested using 50 mM ammonium bicarbonate containing 10 ng/μL trypsin. The digested peptides were sequentially extracted from the pellet using 50% acetonitrile, 5% formic acid, and 100% acetonitrile. They were then separated on an EASY-nLC 1000 ultra-high-performance liquid chromatography system, ionized in an NSI ion source, and analyzed by Thermo Scientific Orbitrap Fusion mass spectrometry. Mass spectrometry sequencing was performed by Jingjie PTM BioLab (Hangzhou) Co., Ltd. Protein interaction data were analyzed using MaxQuant, with common interactors were identified through Venn diagram analysis. Proteins exhibiting a Score > 100 and Intensity > 1 × 10⁶ underwent further characterization via protein–protein interaction network construction using STRING (https://string-db.org) and functional annotation through DAVID (https://david.ncifcrf.gov) for Gene Ontology and pathway enrichment. Final candidate selection prioritized proteins with high composite scores, commercially available antibodies, and functional enrichment in transcriptional regulation, translation, and RNA transport. Selected interactions were validated by co-immunoprecipitation to establish a mechanistic foundation.

### SiRNA transfection experiment

Three pairs of small interfering RNA (siRNA) targeting TAF15 were designed, and all siRNA used in this study were purchased from General Biol (AnHui, China). Three pairs of siRNAs were transfected into A549 cells and the silencing efficiency of each group was verified by RT-qPCR and western blotting, and the siRNA with the highest silencing efficiency was used for subsequent experiments. The selected TAF15 siRNA was transfected into A549 cells overexpressing ROP16-RH, ME49, or VEG, as well as into cells transduced with blank lentiviral vectors, using NC-siRNA as negative controls. The TAF15-siRNA target sequences were listed in Supplementary Table 1.

### Flow cytometry

The A549 cells, A549-HBLV cells, A549-ROP16-RH/ME49/VEG cells, and siRNA-TAF15 transfected cells in logarithmic phase were inoculated in 6-well plates at a density of 5.0 × 10^5^ cells/well and cells were collected after 48 h of incubation. Pre-chilled 75% ethanol was added and cells were fixed overnight at 4 °C. After centrifugation, the supernatant was discarded, and the cells were washed with an appropriate amount of PBS to remove the ethanol. DNA Staining solution and Permeabilization solution were added, and the cells were incubated for 30 min at room temperature, protected from light. The cell cycle was then analyzed using a Flow Cytometer.

Following the manufacturer’s instructions for the Annexin V-APC/7-AAD apoptosis kit, a blank tube was set to adjust the voltage of the FSC, SSC and fluorescence channel on the flow cytometer, and a single staining tube was set up to adjust the fluorescence channel compensation. To the cells in each experimental tube, 5μL Annexin V-APC and 10μL 7-AAD were added. The cells were incubated at room temperature in the dark for 5 min, and the level of apoptosis was analyzed by Flow Cytometry.

### Quantitative real-time polymerase chain reaction analysis

Total RNA was extracted using TRIzol (Invitrogen, USA) and reverse-transcribed to cDNA using a PrimeScript^™^ RT Reagent Kit with gDNA Eraser (Takara, Japan) according to the manufacturer’s instructions. Gene amplification and detection were performed using the LightCycler^®^ 480 System (Roche Diagnostics, Switzerland) and TB Green^®^ Premix Ex Taq^™^ II (Takara, Japan). All transcript levels were normalized to *β*-actin expression. Relative expression was analyzed by the comparative cycle threshold (Ct) method using the equation ${2}^{-\Delta \Delta Ct}$. Primers used in this experiment were synthesized by Shanghai Sangong Biotechnology Co., LTD, with specific sequences shown in Supplementary Table 2.

### Western blotting analysis

The A549 cells, A549-HBLV cells, A549-ROP16-RH/ME49/VEG cells, and siRNA-TAF15 transfected cells were cultured to logarithmic growth phase. Total protein was extracted from each group of cells using the Total Protein Extraction Kit (KeyGEN, China). The concentration of each protein sample was measured by a BCA protein assay kit (KeyGEN, China). The protein samples from each group were supplemented with 5 × Loading Buffer to achieve a final concentration of 1 μg/μL. Subsequently, 10 μL aliquots of each protein sample were subjected to SDS-PAGE electrophoresis. Following separation, proteins were transferred onto PVDF membranes under constant current (300 mA) using an ice-cold transfer system. Antibodies were diluted at ratios ranging from 1:1000 to 1:10,000 in all cases, and membranes were incubated overnight at 4 °C with 50 rpm shaking. The anti-*β*-actin was used as the control. The PVDF membranes were washed with TBST three times, and then incubated with horseradish peroxidase-labeled secondary antibody with gentle shaking for 90 min. Specific bands were visualized using enhanced chemiluminescence. Chemiluminescent signals were detected using western ECL Substrate (Advansta, Menlo Park, CA, USA) and images were captured with a ChemiDoc Imaging System (Bio-Rad, Hercules, CA, USA). Antibodies against 6 × His-Tag (ab18184, 1:2,000), TAF15 (ab134916, 1:10,000), Caspase9 (ab243880, 1:1,000), Bax (ab206292, 1:1,000), Bcl-2 (ab52771, 1:5,000), CDK6 (ab236630, 1:1,000), CyclinD1 (ab59479, 1:1,000), p21(ab109520,1ː5,000), p53 (ab109201, 1:1,000), and *β*-actin (ab179513, 1:1,000) were obtained from Abcam or Proteintech Company, and the secondary antibody (GB23303, 1:8,000) was provided by Proteintech.

### Statistical Analysis

All of the above experiments were performed with three independent biological replicates and technical replicates, and the data were presented as the mean ± standard deviation (SD). Statistical significance was determined using one-way ANOVA with Tukey’s post hoc test for multiple comparisons or Student’s t-test for two-group comparisons. A *p*-value of less than 0.05 was considered statistically significant. GraphPad Prism 8.0 software was used for statistical analysis and graph generation.

## Results

### Establishment of ROP16-overexpressing A549 cell model I\II\III

**Fig. 1:**
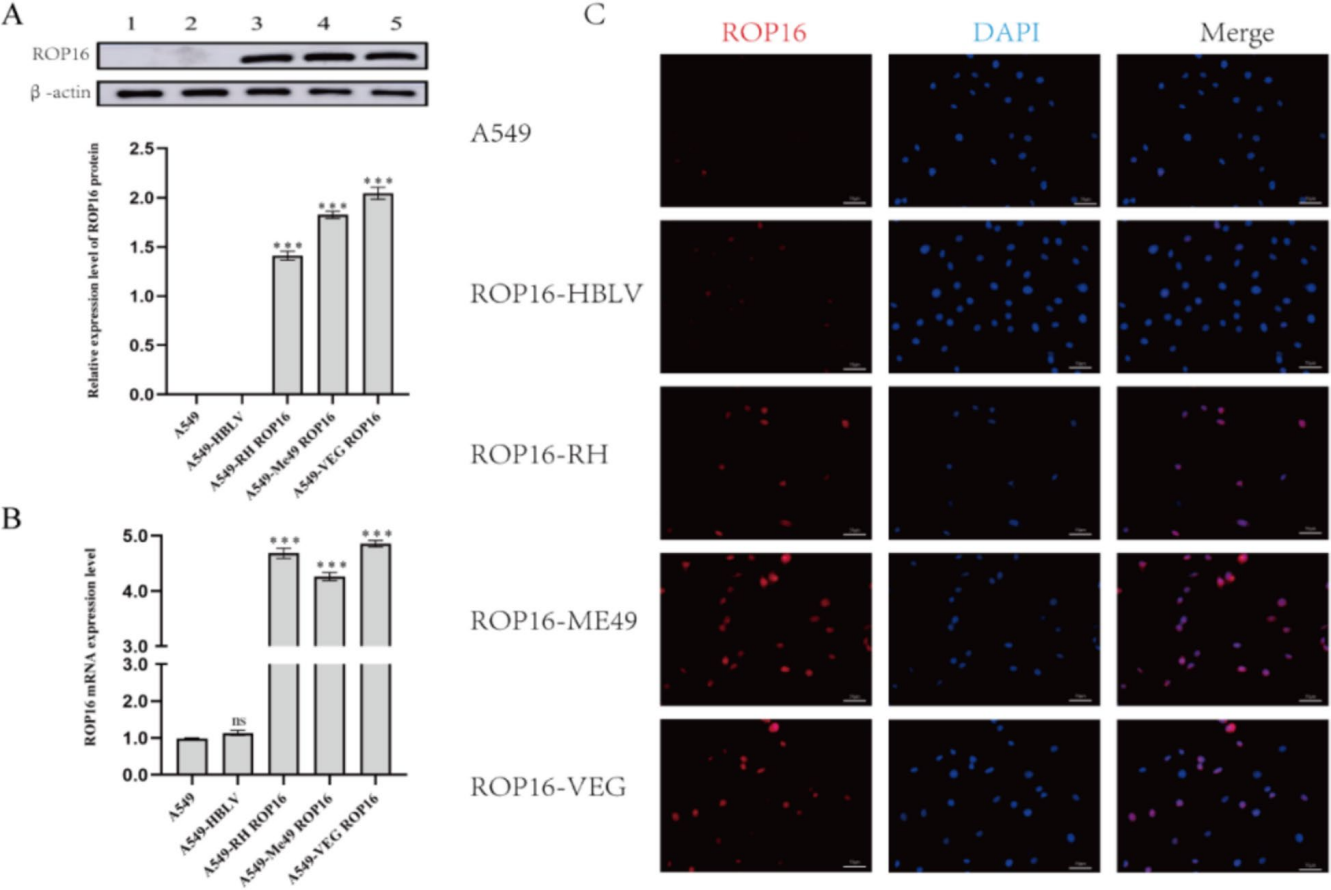
Transfection of A549 cells with lentiviral vector overexpressing ROP16 I/II/III. **A** The expression of ROP16 in A549 was detected by western blotting and the grayscale statistical analysis of this detection results; Lane 1: A549 cells; Lane 2: Negative control cells; Lane 3: A549 cells overexpressing ROP16 I; Lane 4: A549 cells overexpressing ROP16 II; Lane 5: A549 cells overexpressing ROP16 III. Statistical analysis of the grayscale values from the results, the experiments were performed with three independent biological replicates and technical replicates, and the results were expressed as mean ± SD and subjected to one-way ANOVA, compared with the blank group, *** *P* < 0.001. **B** The mRNA expression of ROP16 in A549 was detected by RT-qPCR. The experiments were performed with three independent biological replicates and technical replicates, the data were expressed as mean ± SD and subjected to one-way ANOVA, compared with the blank group, *** *P* < 0.001. **C** Immunofluorescence detection of ROP16 expression in A549 cells (400 ×)

Lentiviral vector of overexpressing *Toxoplasma gondii* ROP16 type I (RH), type II (ME49), and type III (VEG) were constructed using genetic engineering techniques (Supplementary Text 1) and stably transfected into A549 cells. Total RNA and protein were extracted from each group for RT-qPCR and western blotting analysis. Compared with blank controls (A549) and empty vector controls (A549-HBLV), ROP16 (Fig. 1A) and mRNA (Fig. 1B) expression levels were significantly elevated in all ROP16-overexpressing groups. Furthermore, immunofluorescence staining at 72 h post-transfection revealed predominant nuclear localization of ROP16, with partial cytoplasmic expression (Fig. 1C). These findings collectively validate the successful establishment of A549 cell models overexpressing distinct ROP16 genotypes.

### TAF15 is identified as a novel ROP16-interacting protein through Co-IP/MS screening

**Fig. 2:**
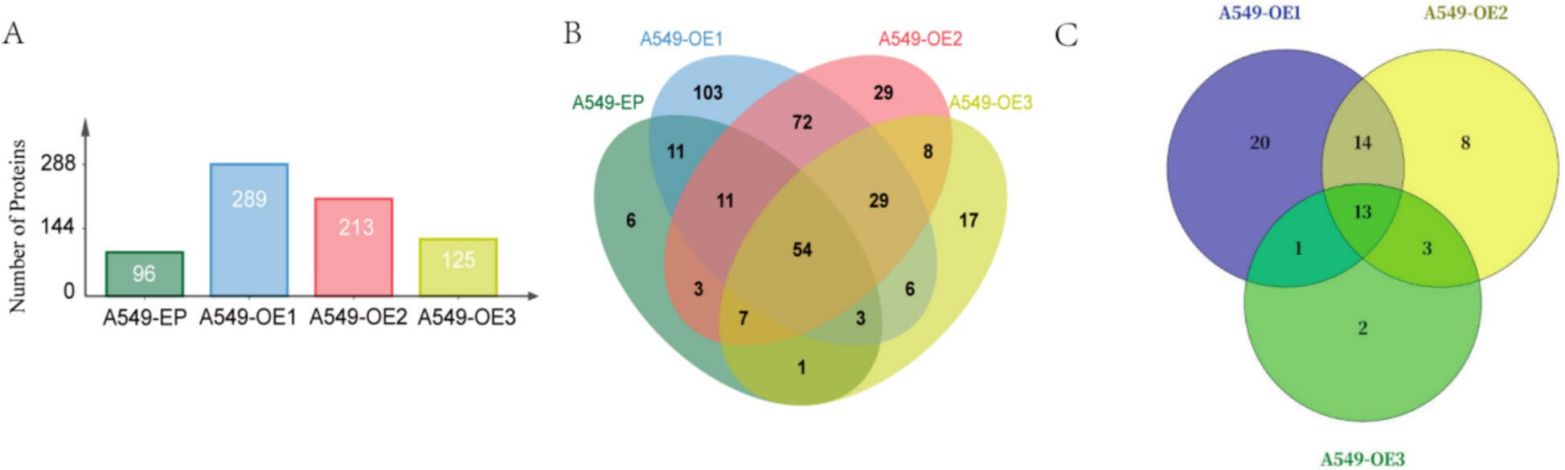
Mass spectrometry analysis of ROP16 interacting proteins. **A** This figure summarizes the quantity of potential interacting proteins present in the three different genotypes of ROP16 compared with the negative control group. **B** The Venn diagram illustrates the distribution of proteins that interact with three different types of ROP16 compared with the negative control group. The numbers in each section of the Venn diagram correspond to the number of potential interacting proteins identified under each specific condition combination. **C** Proteins with a score > 100 were subjected to Venn analysis

**Fig. 3:**
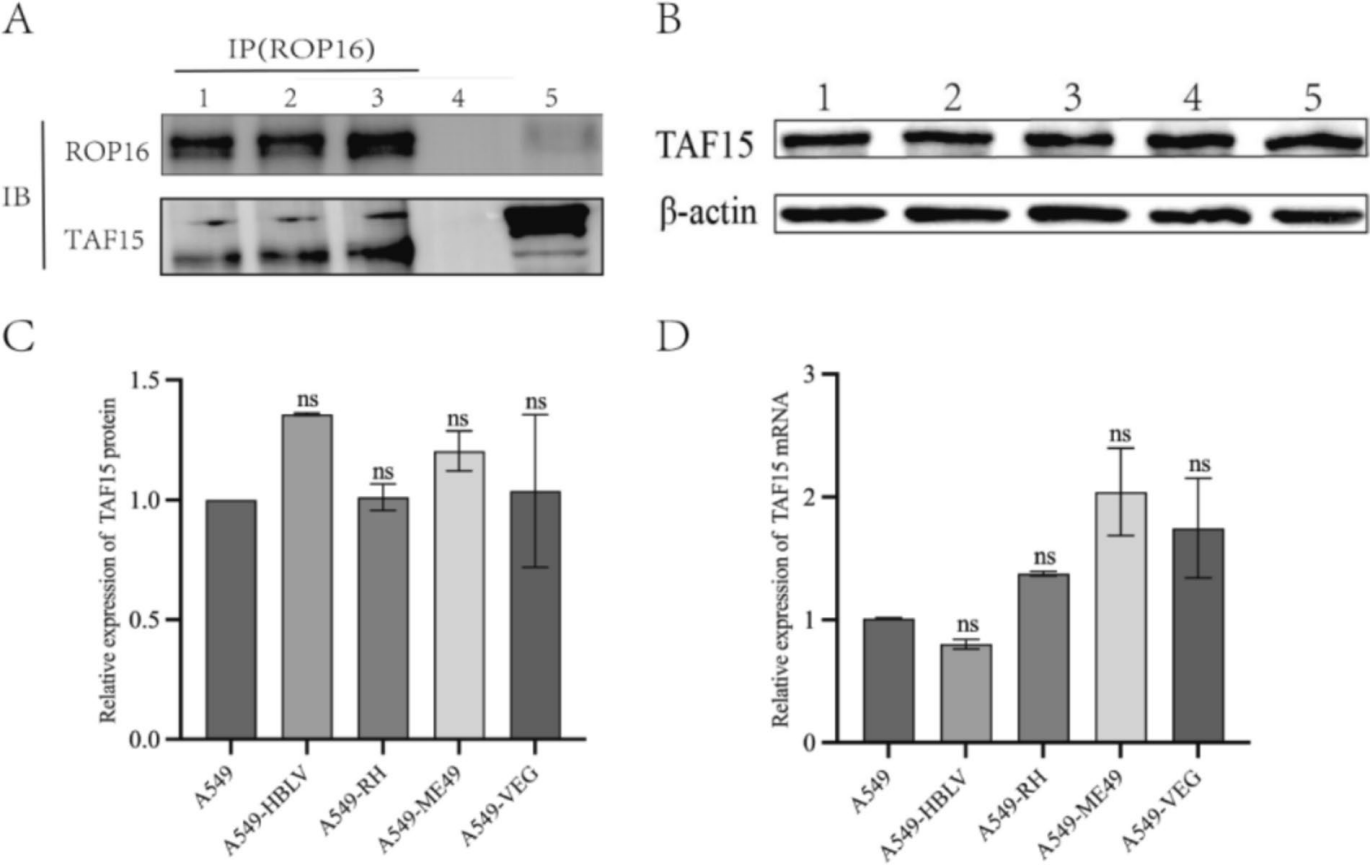
Validation of the interaction between ROP16 and TAF15 and its impact on expression. **A** Co-IP experiments confirm the interaction of TAF15 with different genotypes of ROP16 in A549 cells. Lane 1: A549 cells overexpressing ROP16 I; Lane 2: A549 cells overexpressing ROP16 II; Lane 3: A549 cells overexpressing ROP16 III; Lane 4: Input control; Lane 5: IgG control. **B** Western blotting analysis assessing the effect of overexpressing different types of ROP16 on TAF15 protein expression in A549 cells. Lane 1: A549 cells; Lane 2: Negative control cells; Lane 3: A549 cells overexpressing ROP16 I; Lane 4: A549 cells overexpressing ROP16 II; Lane 5: A549 cells overexpressing ROP16 III. **C** Statistical analysis of the grayscale values from the results in figure (**B)**, the experiments were performed with three independent biological replicates and technical replicates, and the results were expressed as mean ± SD and subjected to one-way ANOVA, compared with the blank group, ns *P* > 0.05. **D** RT-qPCR analysis of the effect of overexpressing different types of ROP16 on the transcription levels of TAF15 in A549 cells, the experiments were performed with three independent biological replicates and technical replicates, and the results were expressed as mean ± SD and subjected to one-way ANOVA, compared with the blank group, ns *P* > 0.05

IP-LC/MS identified potential interacting proteins of type I, II, and III ROP16 in A549 cells. Compared with empty vector-transfected controls, 289, 213, and 125 proteins were specifically identified in A549-RH, A549-ME49, and A549-VEG samples, respectively (Fig. 2A), with 48, 38, and 19 proteins exceeding a Score threshold of 100. Venn analysis revealed 29 shared potential interactors across genotypes (Fig. 2B), including 13 high-confidence interactors (Score > 100) (Fig. 2C). Among these 13 candidates, TAF15 exhibited high binding affinity to ROP16 isoforms and was functionally enriched in transcriptional regulation, RNA transport, and RNA splicing on the basis of GO/pathway analyses. Consequently, ROP16-TAF15 interaction was validated by co-immunoprecipitation (Fig. 3A). Furthermore, western blotting and RT-qPCR confirmed that ROP16 overexpression did not alter endogenous TAF15 expression (Fig. 3B–D).

### Establishment of a ROP16/TAF15 co-engineered A549 model

Three siRNAs (TAF15-288, TAF15-815, TAF15-1812) were constructed and synthesized to silence TAF15, and the silencing effect was verified by western blotting. The results showed that the TAF15 protein level was significantly down-regulated in the siRNA-transfected cells compared with the blank group (Fig. S1A–B), among which siRNA-288 had the highest degree of downregulation. On the basis of the above results, siRNA-288 was selected as the siRNA for silencing TAF15 in the subsequent experiments. Western blotting results showed that the TAF15 protein level was significantly down-regulated in A549 cells overexpressing ROP16 types I, II, and III (Fig. S1C–D). It indicated that transfection of TAF15-siRNA to ROP16 overexpressing A549 cells was successful.

### TAF15 knockdown reverses ROP16-induced apoptosis in a genotype-dependent manner

**Fig. 4:**
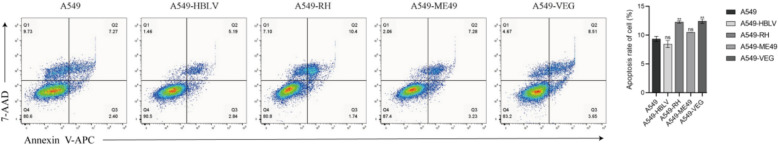
Effect of overexpression of I/II/III ROP16 on apoptosis of A549 cells. Flow cytometry to detect the effect of apoptosis in A549 cells after ROP16 overexpression and apoptosis statistical analysis, the experiments were performed with three independent biological replicates and technical replicates, the results were expressed as mean ± SD and subjected to one-way ANOVA, compared with the blank group, ns *P* > 0.05, ** *P* < 0.01

**Fig. 5:**
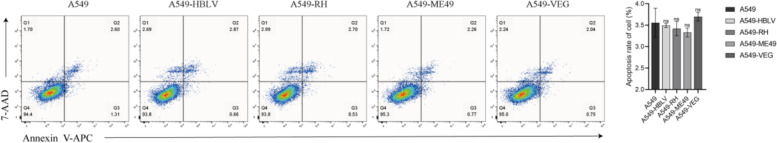
Effect of silencing TAF15 on apoptosis in A549 cells overexpressing I/II/III ROP16. Flow cytometry to detect the effect of silencing TAF15 on apoptosis of A549 cells overexpressing I/II/III ROP16, the experiments were performed with three independent biological replicates and technical replicates, the results were expressed as mean ± SD and subjected to one-way ANOVA, compared with the blank group, ns *P* > 0.05

Flow cytometry analysis of A549 cell apoptosis was performed after 48 h of culture to assess the impact of distinct ROP16 genotypes. Compared with the negative control group, cells expressing type I and III ROP16 exhibited significantly increased apoptosis rates of 12.14% and 12.06%, respectively (*P* < 0.01) (Fig. 4). However, upon TAF15 knockdown, apoptosis levels across all groups showed no significant difference versus controls (Fig. 5).

**Fig. 6:**
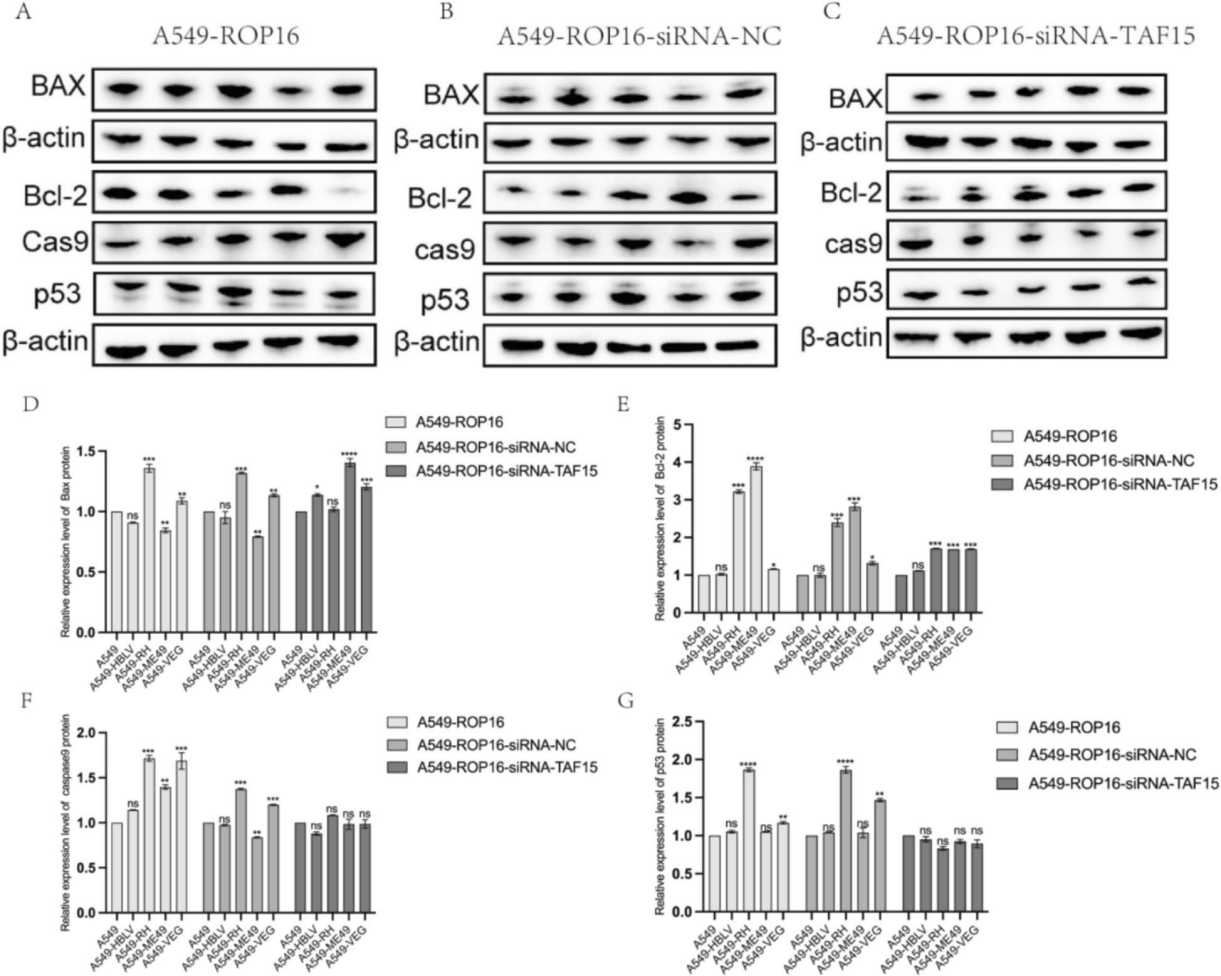
The impact of TAF15 silencing on the expression of apoptosis-related proteins in A549 cells overexpressing I/II/III ROP16. **A** The effects of I/II/III ROP16 on the expression of apoptosis-related proteins in A549 cells. **B** The influence of the siRNA negative control group on the expression of apoptosis-related proteins across the various experimental groups. **C** The effects of silencing TAF15 on the expression of apoptosis-related proteins in A549 cells overexpressing I/II/III ROP16. The lanes are labeled from left to right as follows: blank control group, negative control group, and overexpression groups for ROP16 types I/II/III. **D**–**G** This section presents the statistical analysis of the grayscale values obtained from the western blotting experiments shown in (**A**–**C**), the experiments were performed with three independent biological replicates and technical replicates, the results were expressed as mean ± SD and subjected to one-way ANOVA, compared with the blank group, ns *P* > 0.05; * *P* < 0.05, ** *P* < 0.01, *** *P* < 0.001,****P < 0.0 001

**Fig. 7:**
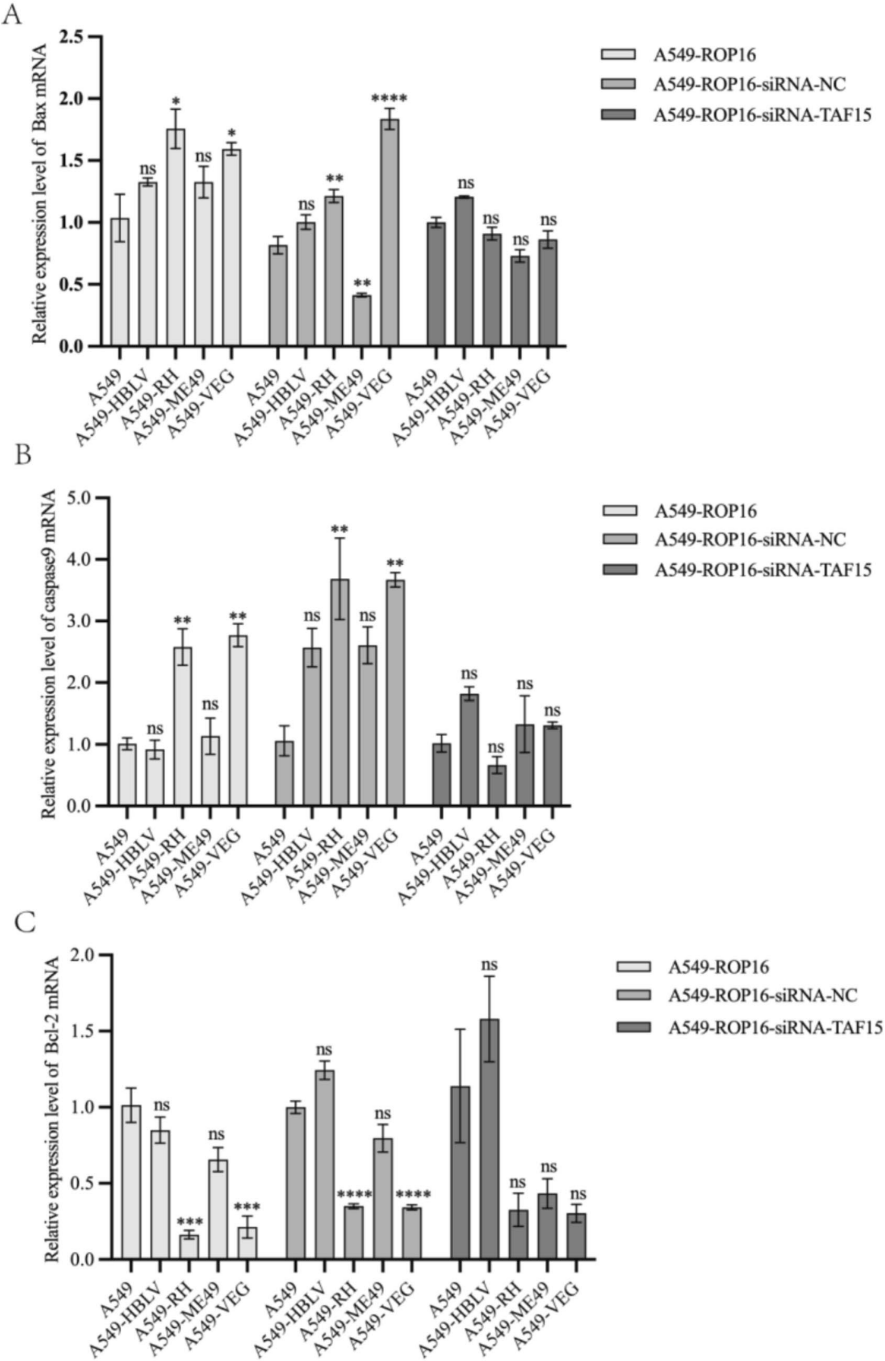
Transcriptome level analysis of apoptosis-related proteins by RT-qPCR. Statistical analysis of the impact of ROP16 on the mRNA expression of Bax, Bcl-2, and Caspase-9 in A549 cells, as well as the changes in this effect following TAF15 protein silencing, the experiments were performed with three independent biological replicates and technical replicates, the results were expressed as mean ± SD and subjected to one-way ANOVA, compared with the blank group, ns *P* > 0.05; * *P* < 0.05, ** *P* < 0.01, *** *P* < 0.001,**** *P* < 0.0 001

To further investigate ROP16-mediated apoptotic regulation, western blotting and RT-qPCR were employed to quantify expression of apoptosis-related proteins (BAX, BCL-2, Caspase 9, p53). In A549 cells overexpressing type I/III ROP16, pro-apoptotic BAX was significantly upregulated while anti-apoptotic BCL-2 was downregulated, accompanied by elevated p53 and Caspase 9 expression. These findings demonstrate the pro-apoptotic function of type I/III ROP16, contrasting with the negligible impact of type II ROP16 (Fig. 6A). Following TAF15 silencing in ROP16-overexpressing cells, apoptotic protein expression was re-evaluated. First, all cell groups were transfected with negative control siRNA (siRNA-NC) and analyzed by western blotting to exclude potential non-specific effects on apoptosis (Fig. 6B). Subsequently, pre-selected siRNA was transfected into ROP16-overexpressing groups under identical conditions. Comparative analysis revealed significantly downregulated BAX expression in type I/III versus type II ROP16-expressing cells (*P* < 0.05). No statistically significant differences were observed in BCL-2, Caspase 9, or p53 levels across genotypes (Fig. 6C). Consistent with protein data, RT-qPCR analysis of mRNA expression confirmed that TAF15 knockdown selectively attenuated the pro-apoptotic effects of type I/III ROP16 isoforms (Fig. 7).

To investigate the cause of this discrepancy, we further analyzed the differences in the gene and amino acid sequences of ROP16 secreted by different *Toxoplasma gondii* genotypes. The results demonstrated that the nucleotide sequences of type I and type III exhibited a high degree of similarity (> 99%), whereas the similarity between type II and type I/III sequences was approximately 97%. Furthermore, type I and type III differed at only a single amino acid position (position 661), in contrast to the over thirty amino acid differences observed between type I/III and type II. It is likely that this high degree of sequence similarity between type I and type III accounts for their shared functional characteristics (Fig. S2–S3).

### TAF15 depletion disrupts ROP16-driven cell cycle in a genotype-dependent manner

**Fig. 8:**
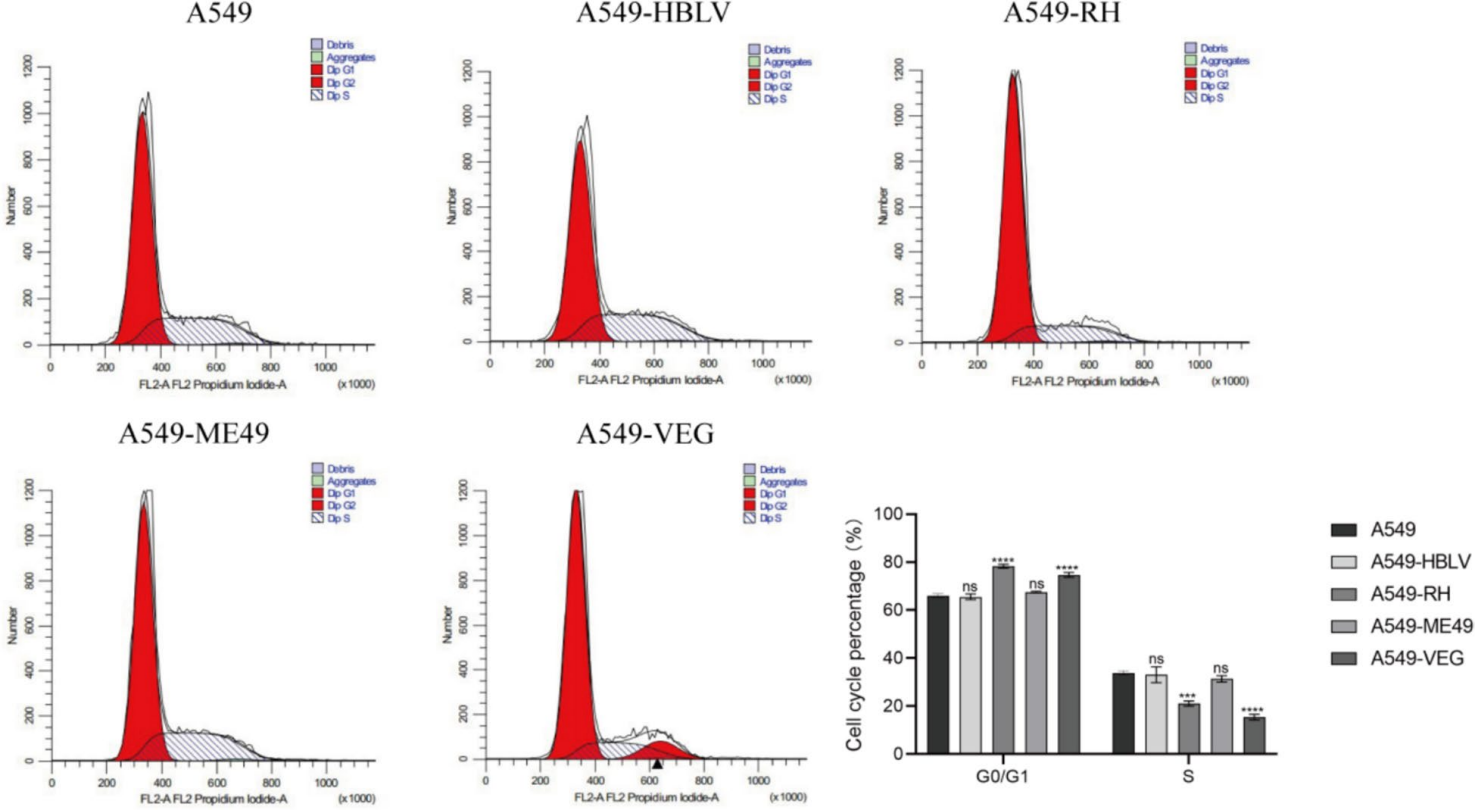
Effect of overexpression of I/II/III ROP16 on A549 cell cycle. Flow cytometry to detect the effect of A549 cell cycle after ROP16 overexpression and statistical analysis of apoptosis, the experiments were performed with three independent biological replicates and technical replicates, the results were expressed as mean ± SD and subjected to one-way ANOVA, compared with the blank group, ns *P* > 0.05, *** *P* < 0.001,**** *P* < 0.0001

**Fig. 9:**
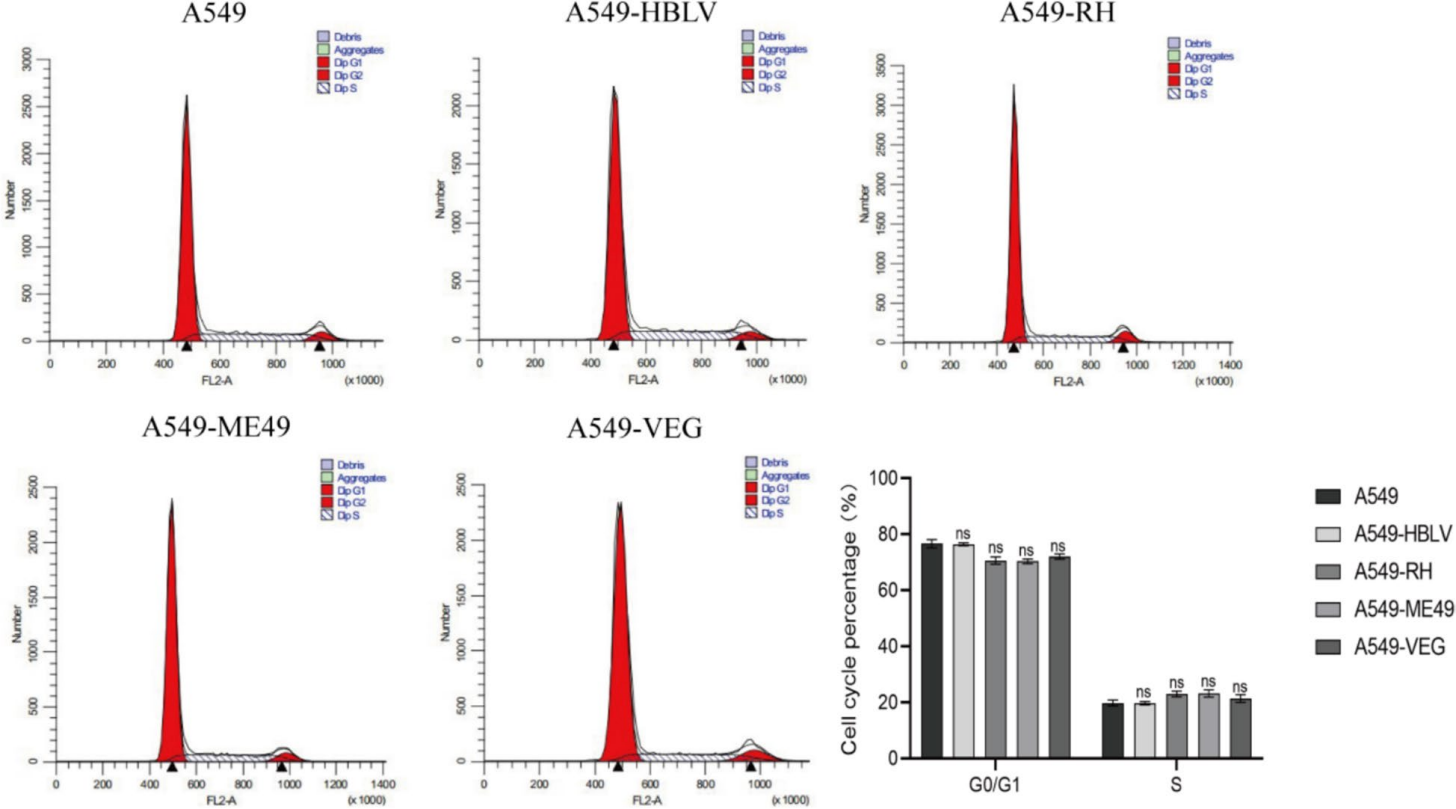
Effect of silencing TAF15 on the cell cycle of A549 cells overexpressing I/II/III ROP16. Flow cytometry detection of the effect of silencing TAF15 on the cell cycle of A549 cells overexpressing I/II/III ROP16 and statistical analysis of the cycle, the experiments were performed with three independent biological replicates and technical replicates, the results were expressed as mean ± SD and subjected to one-way ANOVA, compared with the blank group, ns *P* > 0.05

**Fig. 10:**
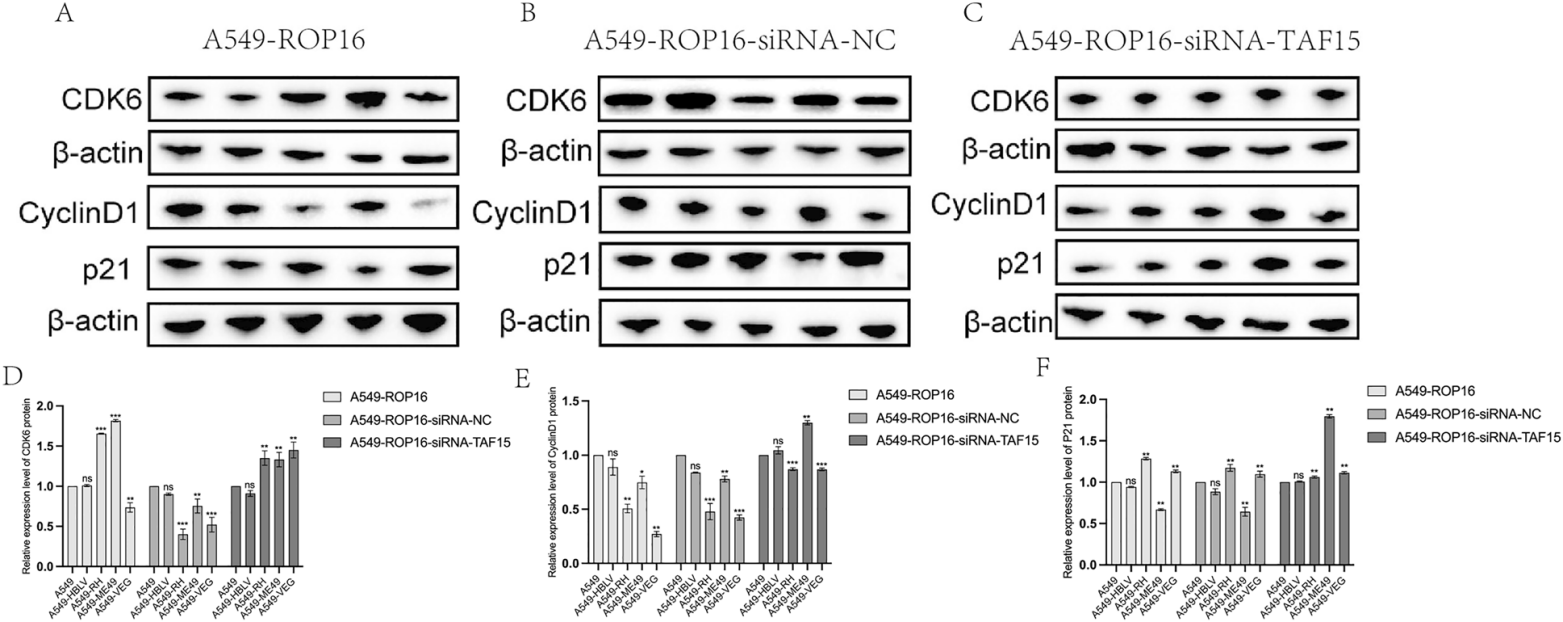
The impact of TAF15 silencing on the expression of cell cycle-related proteins in A549 Cells overexpressing I/II/III ROP16. The **A**–**C** graphs respectively represent the effects of cell cycle-related protein expression in A549 cells overexpressing I/II/III ROP16, cells transfected with siRNA negative control group, and cells silencing TAF15 in each group. In the western blotting experiment, the lanes from left to right were respectively marked as: blank control group, negative control group, and overexpression group I/II/III. **D**–**F** This section presents the statistical analysis of the grayscale values obtained from the western blotting experiments shown in (**A**–**C**), the experiments were performed with three independent biological replicates and technical replicates, the results were expressed as mean ± SD and subjected to one-way ANOVA, compared with the blank group, ns *P* > 0.05; * *P* < 0.05, ** *P* < 0.01, *** *P* < 0.001,**** *P* < 0.0 001

Previous studies suggest that specific *Toxoplasma gondii* ROP16 genotypes may regulate cell cycle progression. Here, we assessed the impact of distinct ROP16 genotypes on the A549 cell cycle using flow cytometry. The results indicate that the proportion of G0/G1 phase cells overexpressing type I ROP16 cells increased from 65.40% to 78.39% (*P* < 0.0001), and the proportion of S phase cells decreased from 34.38% to 21.34% (*P* < 0.001). The proportion of G0/G1 phase cells overexpressing type III ROP16 cells increased to 75.40% (*P* < 0.0001), and the S phase decreased to 14.07% (*P* < 0.0001) (Fig. 8). While knockdown of TAF15 showed no significant difference in the cell cycle of each group compared with the control group (Fig. 9). Subsequently, we performed western blotting analysis to assess the expression levels of G1 phase-related proteins—p21, CDK6, and Cyclin D1—in the different groups of A549 cells (Fig. 10A). The results indicated that, compared with the blank control group, the overexpression of type I and type III ROP16 led to an upregulation of p21 and CDK6 protein expression, while Cyclin D1 were significantly downregulated. These findings suggest that type I and type III ROP16 have an inhibitory effect on the cell cycle of A549 cells.

Following this, we examined the expression levels of cell cycle-related proteins after silencing siRNA-NC and siRNA-TAF15 using the same method. The results showed that, compared with the non-transfected group, the transfection of siRNA-NC had no significant effect on the expression of the proteins across the groups (Fig. 10B). However, in the siRNA-TAF15 transfection group, CDK6 protein expression was upregulated after TAF15 silencing compared with the negative control group, but no statistical differences were observed among the three genotypes of ROP16 overexpression groups (Fig. 10C). Comparative analysis of Cyclin D1 expression levels among the overexpression group, siRNA-NC group, and siRNA-TAF15 group indicated that TAF15 silencing did not affect the inhibitory effect of type I and type III ROP16 proteins on Cyclin D1 expression (Fig. 10D–E). Additionally, in the type II ROP16 overexpression group, the originally downregulated p21 protein was found to be upregulated after TAF15 silencing, while the levels in the type I and type III ROP16 groups remained elevated. Western blotting analysis demonstrate that TAF15 silencing partially attenuated the cell cycle inhibitory effects in cells overexpressing type I/III ROP16 (Fig. 10F).

**Fig. 11:**
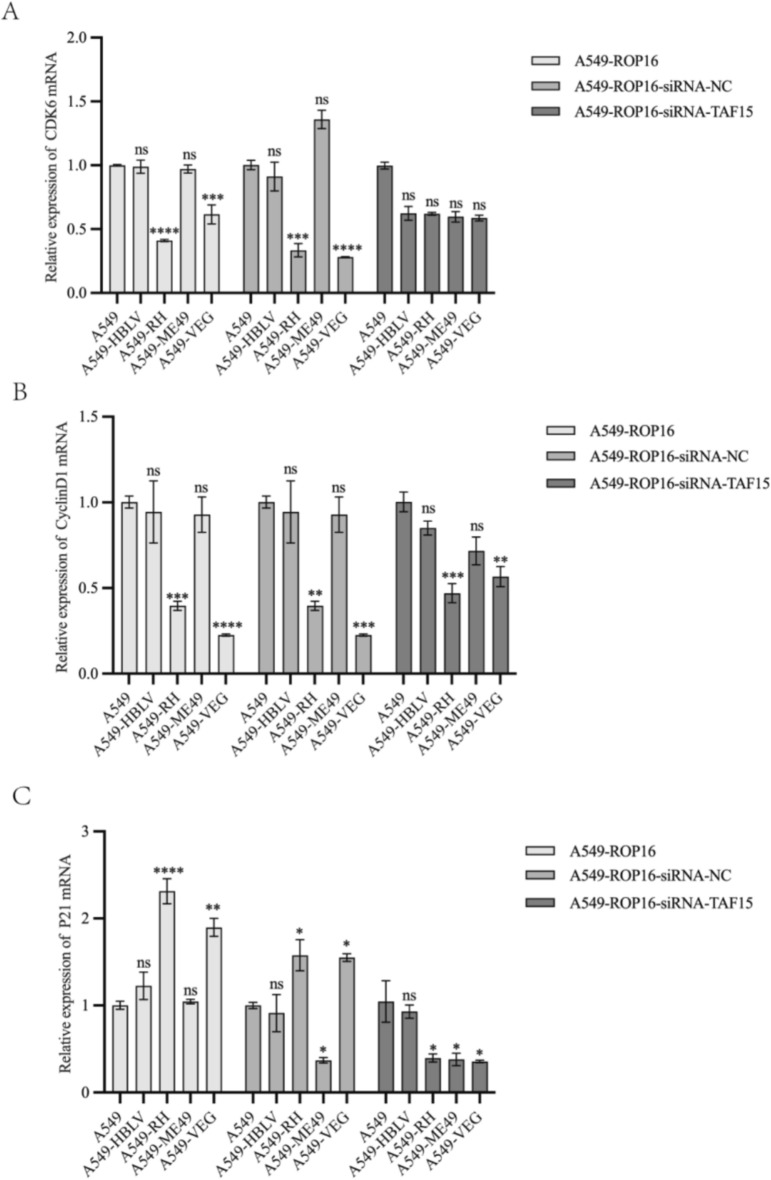
Transcriptome level analysis of cell cycle-related proteins by RT-qPCR. Statistical analysis of the impact of ROP16 on the mRNA expression of CDK6, CyclinD1 and p21 in A549 cells, as well as the changes in this effect following TAF15 protein silencing, the experiments were performed with three independent biological replicates and technical replicates, the results were expressed as mean ± standard deviation and subjected to one-way ANOVA, compared with the blank group, ns *P* > 0.05; * *P* < 0.05, ** *P* < 0.01, *** *P* < 0.001,**** *P* < 0.0 001

Additionally, total RNA was extracted from each group of cells and used to assess the mRNA expression levels of p21, CDK6, and Cyclin D1 via RT-qPCR. The results for p21 and Cyclin D1 were in agreement with those obtained from western blotting analysis. However, CDK6 exhibited significant downregulation at the transcriptional level while showing upregulation at the protein level, suggesting potential involvement of post-transcriptional regulation (Fig. 11). Collectively, the vast majority of concordant regulation observed at both the mRNA and protein levels further confirms the modulatory effect of type I and type III ROP16 on the A549 cell cycle via TAF15.

## Discussion

Extensive scientific evidence from human studies, animal models, and cells experiments indicates that parasites have significant anti-tumor effects. The exploration of parasite-derived proteins as anti-tumor agents has become a research hotspot. One promising direction is the utilization of unique functional proteins secreted by parasites for anti-tumor therapy [1, 3].

ROP16 is a protein secreted by *Toxoplasma gondii* during host cell invasion, characterized by a serine/threonine (S/T) kinase domain [26]. Research indicates that ROP16 can enter the host cell nucleus within 10 min post-infection, exhibiting a strong fluorescent signal detectable for up to 4 h and persisting for as long as 24 h. This rapid nuclear entry enables ROP16 to significantly influence host cell proliferation, differentiation, and apoptosis, while also participating in the transcription and translation of various signaling pathways [20, 27]. The gene sequence of ROP16 varies among different *Toxoplasma* strains, thereby regulating multiple signaling pathways and gene transcription levels in infected host cells [28]. Notably, the NF-κB pathway, which is crucial for regulating inflammatory, immune, and anti-apoptotic responses, is influenced by ROP16, as it has been shown to inhibit NF-κB activation in host cells [29]. Additionally, the JAK-STAT signaling pathway, which plays a key role in cell proliferation, differentiation, apoptosis, and immune response regulation, is activated by ROP16 during *Toxoplasma gondii* invasion. Specifically, ROP16 activates JAK kinase, leading to the phosphorylation of STAT proteins. These phosphorylated STAT proteins undergo conformational changes, become activated, and translocate to the nucleus, further modulating host cell responses [26]. Additionally, ROP16 can polyubiquitinate STING, inhibiting the cGAS-STING signaling pathway, reducing inflammatory cytokine secretion, and inhibiting STAT1 and NF-κB pathways, ultimately preventing brain metastasis of breast and lung cancer. *Toxoplasma gondii*-infected dendritic cells (DCs) exosomes (Me49-DC-Exo) promote M1 macrophage polarisation by regulating macrophage polarisation and inhibit M2 macrophage polarisation of SOCS1 by its enriched miR-155-5p target cytokine signalling suppressor 1 (SOCS1), thereby enhancing anti-tumour immunity and inhibiting colorectal cancer tumour growth [30].

The results of this study demonstrate that type I and III ROP16 significantly induce apoptosis and cell cycle arrest in A549 lung adenocarcinoma cells by regulating cell cycle-related proteins (such as p21, CDK6, and Cyclin D1) as well as apoptosis-related proteins (including Bax, BCL-2, p53, and Caspase-9). In contrast, type II ROP16 did not exhibit similar effects. This finding suggests that the function of ROP16 is type-specific, which may be attributed to structural differences among the ROP16 types or the specificity of their interactions with host cell proteins. On the other hand, the lack of such motifs in type II ROP16 could explain its inability to induce similar biological effects. Previous studies have indicated that type I and type III ROP16 can phosphorylate STAT3/6, thereby activating the JAK-STAT signaling pathway and altering cellular survival outcomes. In contrast, type II ROP16 lacks this functionality. This functional disparity is likely attributable to a critical amino acid difference at position 503 between type I/III and type II ROP16 [27]. The underlying cause of this functional difference is most likely rooted in this specific amino acid variation and the distinct structural features it confers. These findings provide crucial insights for the future engineering of ROP16.

A limitation of this study is that it was conducted solely in the A549 cell line, without validation in in vivo models or other cancer types. Future research should further investigate the specific molecular mechanisms of the ROP16-TAF15 interaction and evaluate its potential as a therapeutic target for lung cancer. For instance, CRISPR/Cas9 technology could be employed to knockout the TAF15 gene, allowing for more precise validation of TAF15 role in ROP16-mediated apoptosis and cell cycle regulation. Additionally, future studies should validate the biological effects of the ROP16-TAF15 interaction in animal models and explore its universality across other cancer types.

TAF15 (TATA-box binding protein associated factor 15 is an RNA-binding protein from the FET (FUS, EWS, TAF15) family. It plays a key role in gene expression regulation, shuttling within the nucleus, cytoplasm, and cell membrane surface. TAF15 functions include polyadenylation, capping, RNA splicing, modification, localization, export, translation, and transport [24, 25]. Its unique charge distribution enhances interaction with the C-terminal domain of RNA polymerase II, suggesting a strong role in promoting gene transcription and expression. TAF15 can participate directly or indirectly in the assembly of TFIID and act as a coactivator of the transcription initiation complex [31], regulating cell viability by influencing genes related to the cell cycle and apoptosis [25]. M Ballarino et al. found that high TAF15 levels are needed for rapid cellular proliferation and TAF15 depletion had a growth-inhibitory effect and resulted in increased apoptosis [32]. TAF15 is able to regulate cell cycle by regulating the expression of genes related to cell cycle such as CDK6 [32]. In this research, similarly, the expression levels of the genes involved in the cell cycle, such as CDK6, also downregulated significantly. Overexpression of TAF15 in NSCLC correlates with poorer patient survival. Studies by Singh et al. [25]. Indicate that silencing TAF15 significantly reduces NSCLC cell proliferation, induces cell cycle arrest, and promotes apoptosis. Additionally, Su et al. found that TIFIγ inhibits TAF15/TBPmediated IL-6 transcriptional activation, competing with TAF15 for TBP binding and modifying TAF15 through multiple monoubiquitinations [33], this process inhibits epithelial-mesenchymal transformation and metastasis in lung adenocarcinoma. GMDS-AS1 stabilizes SIRT1 mRNA by recruiting TAF15, leading to p65 deacetylation and reduced MMP-9 expression, acting as a tumor suppressor in LUAD [34]. On the basis of its functions such as RNA transport, TAF15 plays an important role in cell growth. In this study, we pay more attention to the effect of TAF15 silencing on the functions of different genotypes of ROP16 to clarify part of the mechanism by which ROP16 inhibits the malignant biological behavior of lung adenocarcinoma cells.

In this study, we identified possible interaction proteins of ROP16 types I, II, and III in A549 cells using immunoprecipitation coupled with IP-LC/MS and bioinformatics analysis. A total of 29 possible interaction proteins were identified in the ROP16 overexpression group. Further analysis showed high relative scores and intensities for TAF15 binding to different ROP16 types. Immunoprecipitation confirmed significant interactions between TAF15 and the three ROP16 types. Further research revealed that TAF15 plays a critical role in ROP16-mediated apoptosis and cell cycle regulation. Upon TAF15 silencing, the pro-apoptotic and cell cycle arrest effects induced by type I and III ROP16 were significantly attenuated. This indicates that the function of ROP16 is dependent on its interaction with TAF15, suggesting that TAF15 may serve as a key mediator in the downstream signaling pathways of ROP16, providing new avenues for exploring the molecular mechanisms underlying the ROP16-TAF15 interaction.

## Conclusions

This study elucidates the differential effects of *Toxoplasma gondii* ROP16 genotypes on cellular processes in A549 lung adenocarcinoma cells. Crucially, TAF15 silencing abolishes the tumor-suppressive functions of type I/III ROP16, establishing TAF15 as a key mediator in ROP16-induced oncosuppression. These findings not only shed new light on the anti-tumor mechanisms of parasite-derived proteins through precise targeting of host RNA-binding machinery, but also provide novel insights into the pathophysiological interplay between microbial effectors and cancer-associated RNA regulatory networks.

## Supplementary Information

Supplementary material 1.

Supplementary material 2.

Supplementary material 3.

Supplementary material 4.

## Data Availability

Data supporting the main conclusions of this study are included in the manuscript.
