# Supplementary material for: TAF15 mediates ROP16-induced apoptosis and cell cycle arrest in lung cancer
Source: Parasit Vectors. 2025 Jul 19;18:287. doi: 10.1186/s13071-025-06933-6 (PMC12276700; doi:10.1186/s13071-025-06933-6)
Supplement: Supplementary file 1 — Supplementary material 1. [file 13071_2025_6933_MOESM1_ESM.docx]

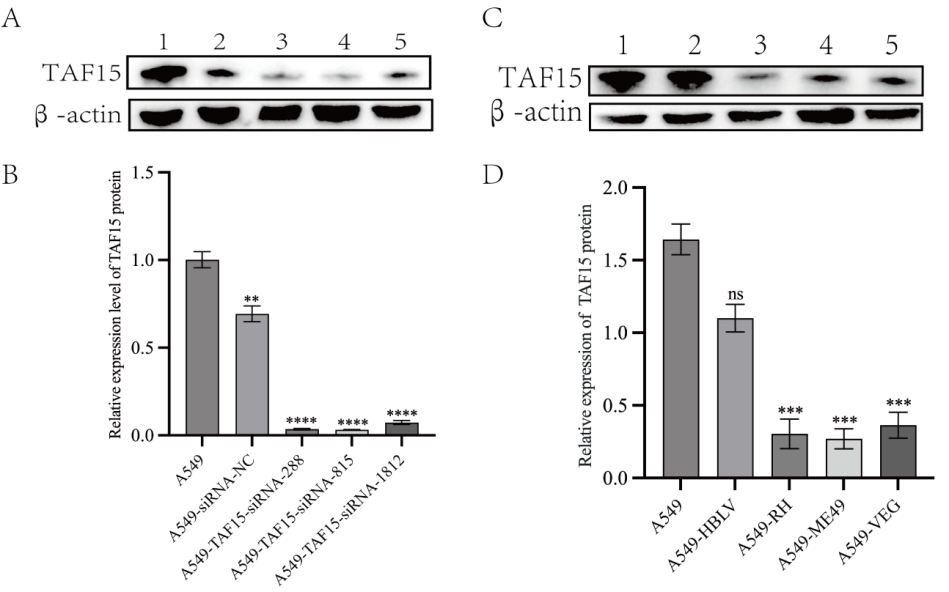


Supplementary Fig. 1

**Supplementary Fig. 1** Construction of TAF15 expression silencing cell model

1. Western blotting analysis of the silencing efficiency of three siRNAs targeting TAF15. Lanes 1-5 represent: blank control group, negative control group, and three different siRNAs. (B)Statistical analysis of the grayscale values corresponding to the results shown in figure A. The experiments were performed with three independent biological replicates and technical replicates, the results were expressed as mean ± SD and subjected to one-way ANOVA, compared with the blank group, ** *P* < 0.01, *** *P* < 0.001. (C)Western blotting analysis of the silencing efficiency of siRNA-TAF15 in A549 cells overexpressing different types of ROP16. Lanes 1-5 represent: blank control group, negative control group, and groups overexpressing ROP16 genotypes I, II, and III, respectively. (D)Statistical analysis of the grayscale values corresponding to the results shown in figure C. The experiments were performed with three independent biological replicates and technical replicates, the results were expressed as mean ± SD and subjected to one-way ANOVA, compared with the blank group, ns *P* > 0.05, *** *P* < 0.001.


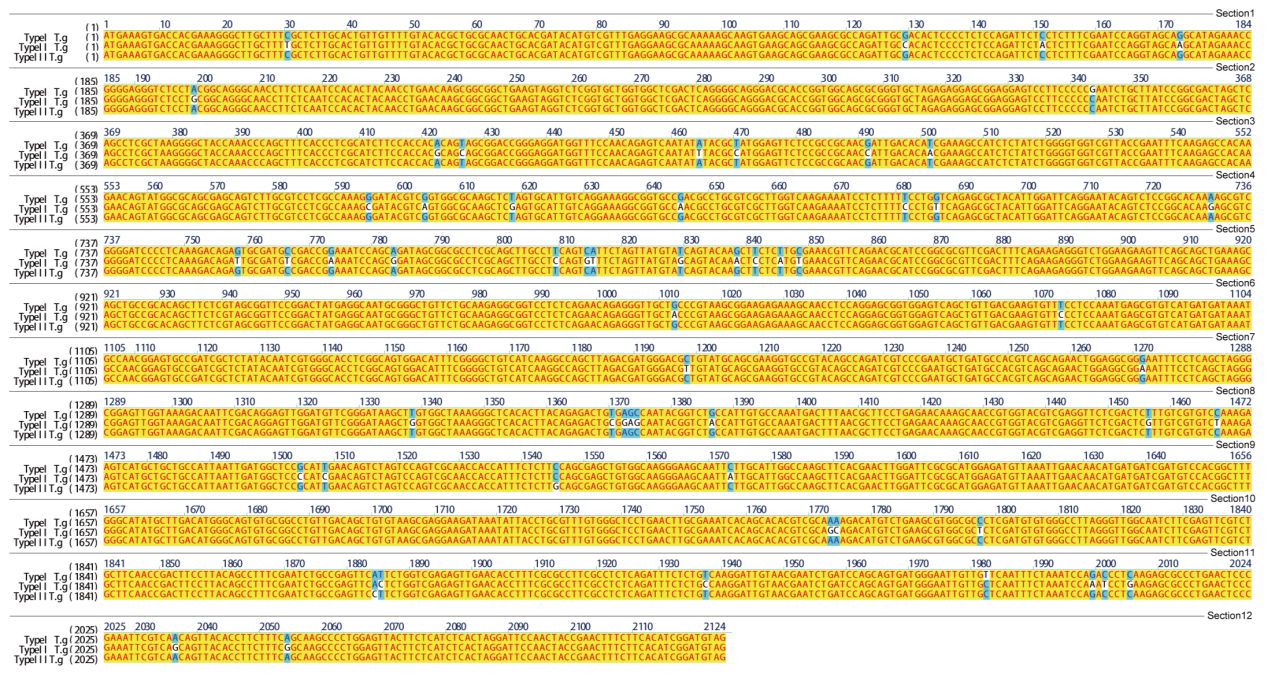


Supplementary Fig. 2

**Supplementary Fig. 2** Sequence difference maps of ROP16 protein genes of three different genotypes

Note:Yellow section: identical genes in three different genotypes of ROP16 protein; Blue section: differential genes in three different genotypes of ROP16 proteins


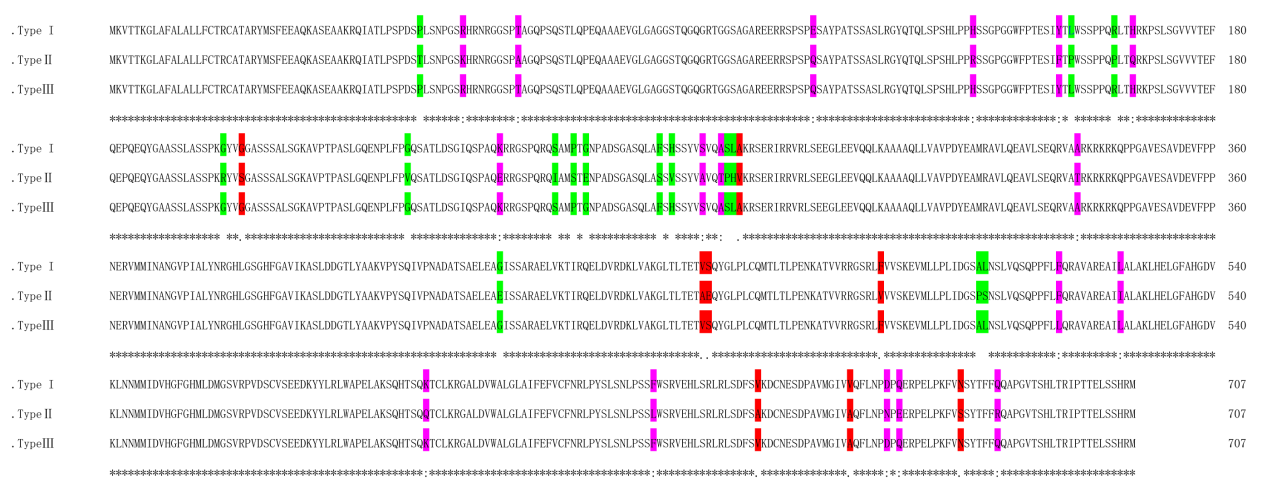


Supplementary Fig. 3

**Supplementary Fig. 3** Amino acid sequence difference maps of ROP16 protein of three different genotypes

Note:Colored sections indicate differential genes
