# Supplementary material for: TAF15 mediates ROP16-induced apoptosis and cell cycle arrest in lung cancer
Source: Parasit Vectors. 2025 Jul 19;18:287. doi: 10.1186/s13071-025-06933-6 (PMC12276700; doi:10.1186/s13071-025-06933-6)
Supplement: Supplementary file 2 — Supplementary material 2. [file 13071_2025_6933_MOESM2_ESM.docx]

Supplementary Table 1.TAF15-siRNA target sequences

| Gene names | Forward Primer Sequence（5’-3’） | Reverse Primer Sequence（5’-3’） |
| --- | --- | --- |
| TAF15siRNA-288 | GGGUGUGUCUACAGAUCAATT | UUGAUCUGUAGACACACCCTT |
| TAF15siRNA-815 | GGUGGUUAUGAGAAUCAAATT | UUUGAUUCUCAUAACCACCTT |
| TAF15siRNA-1218 | GGAAGAAACGACUACAGAATT | UUCUGUAGUCGUUUCUUCCTT |
| siRNA-NC | UUCUCCGAACGUGUCACGUTT | ACGUGACACGUUCGGAGAATT |
