# Supplementary material for: TAF15 mediates ROP16-induced apoptosis and cell cycle arrest in lung cancer
Source: Parasit Vectors. 2025 Jul 19;18:287. doi: 10.1186/s13071-025-06933-6 (PMC12276700; doi:10.1186/s13071-025-06933-6)
Supplement: Supplementary file 3 — Supplementary material 3. [file 13071_2025_6933_MOESM3_ESM.docx]

Supplementary Table 2.qRT- PCR primer sequences

| Gene names | Sequences |
| --- | --- |
| *rop*16(F) | 5´-TGGGCTCCTGAACTTGCGAAATC-3´ |
| *rop*16(R) | 5´-AGACGAACTCGAAGATTGCCAACC-3´ |
| Bax(F) | 5´-ATCAGAACCATCATGGGCTGGACA-3 |
| Bax(R) | 5´-AGCCCATCTTCTTCCAGATGGTGA-3´ |
| Bcl-2(F) | 5´-TTGTGGCCTTCTTTGAGTTCGGTG-3´ |
| Bcl-2(R) | 5´-ACTCACATCACCAAGTGCACCTAC-3´ |
| Caspase9(F) | 5´-TGGTGCTCAGACCAGAGATT-3´ |
| Caspase9(R) | 5´-ACGGGGTGGCATCTGGCTCG-3´ |
| p21(F) | 5´-GTCACTGTCTTGTACCCTTGTG-3´ |
| p21(R) | 5´-GGCGTTTGGAGTGGTAGAAA-3´ |
| CDK6(F) | 5´-TGCACAGTGTCACGAACAGACAGA-3´ |
| CDK6(R) | 5´-TTAGATCGCGATGCACTACTCGGT-3´ |
| CyclinD1(F) | 5´-CAAGCTCAAGTGGAACCTGG-3´ |
| CyclinD1(R) | 5´-GCGGATGATCTGTTTGTTCT-3´ |
| β-actin(F) | 5´-CACTGTGCCCATCTACGA-3´ |
| β-actin(R) | 5´-TGATGTCACGCACGATTT-3´ |
