# Supplementary material for: TAF15 mediates ROP16-induced apoptosis and cell cycle arrest in lung cancer
Source: Parasit Vectors. 2025 Jul 19;18:287. doi: 10.1186/s13071-025-06933-6 (PMC12276700; doi:10.1186/s13071-025-06933-6)

**Results of sequencing:**

**ROP16-RH：**

TGTCGTAACAACTCCGCCCCATTGACGCAAATGGGCGGTAGGCGTGTACGGTGGGAGGTCTATATAAGCAGAGCTCGTTTAGTGAACCGTCAGATCGCCTGGAGACGCCATCCACGCTGTTTTGACCTCCATAGAAGACACCGACTCTACTAGAGGATCTATTTCCGGTGAATTCGCCACCATGCATCATCACCATCACCATAAAGTGACCACGAAAGGGCTTGCTTTCGCTCTTGCACTGTTGTTTTGTACACGCTGCGCAACTGCACGATACATGTCGTTTGAGGAAGCGCAAAAAGCAAGTGAAGCAGCGAAGCGCCAGATTGCGACACTCCCCTCTCCAGATTCTCCTCTTTCGAATCCAGGTAGCAGGCATAGAAACCGGGGAGGGTCTCCTACGGCAGGGCAACCTTCTCAATCCACACTACAACCTGAACAAGCGGCGGCTGAAGTAGGTCTCGGTGCTGGTGGCTCGACTCAGGGGCAGGGACGCACCGGTGGCAGCGCGGGTGCTAGAGAGGAGCGGAGGAGTCCTTCCCCCGAATCTGCTTATCCGGCGACTAGCTCAGCCTCGCTAAGGGGCTACCAAACCCAGCTTTCACCCTCGCATCTTCCACCACACAGTAGCGGACCGGGAGGATGGTTTCCAACAGAGTCAATATATACGCTATGGAGTTCTCCGCCGCAACGATTGACACATCGAAAGCCATCTCTATCTGGGGTGGTCGTTACCGAATTTCAAGAGCCACAAGAACAGTATGGCGCAGCGAGCAGTCTTGCGTCCTCGCCAAAGGGATACGTCGGTGGCGCAAGCTCTAGTGCATTGTCAGGAAAGGCGGTGCCGACGCCTGCGTCGCTTGGTCAAGAAAATCCTCTTTTTCCTGGTCAGAGCGCTACATTGGATTCAGGAATACAGTCTCCGGCACAAAAGCGTCGGGGATCCCCTCAAAGACAGAGTGCGATGCCGACCGGAAATCCAGCAGATAGCGGCGCCTCGCAGCTTGCCTTCAGTCATTCTAGTTATGTATCAGTACAAGCTTCTCTTGCGAAACGTTCAGAACGCATCCGGCGCGTTCGACTTTCAGAAGAGGGTCTGGAAGAAGTTCAGCAGCTGAAAGCAGCTGCCGCACAGCTTCTCGTAGCGGTTCCGGACTATGAGGCAATGCGGGCTGTTCTGCAAGAGGCGGTCCTCTCAGAACAGAGGGTTGCTGCCCGTAAGCGGAAGAGAAAGCAACCTCCAGGAGCGGTGGAGTCAGCTGTTGACGAAGTGTTTCCTCCAAATGAGCGTGTCATGATGATAAATGCCAACGGAGTGCCGATCGCTCTATACAATCGTGGGCACCTCGGCAGTGGACATTTCGGGGCTGTCATCAAGGCCAGCTTAGACGATGGGACGCTGTATGCAGCGAAGGTGCCGTACAGCCAGATCGTCCCGAATGCTGATGCCACGTCAGCAGAACTGGAGGCGGGAATTTCCTCAGCTAGGGCGGAGTTGGTAAAGACAATTCGACAGGAGTTGGATGTTCGGGATAAGCTTGTGGCTAAAGGGCTCACACTTACAGAGACTGTGAGCCAATACGGTCTGCCATTGTGCCAAATGACTTTAACGCTTCCTGAGAACAAAGCAACCGTGGTACGTCGAGGTTCTCGACTCTTTGTCGTGTCCAAAGAAGTCATGCTGCTGCCATTAATTGATGGCTCCGCATTGAACAGTCTAGTCCAGTCGCAACCACCATTTCTCTTCCAGCGAGCTGTGGCAAGGGAAGCAATTCTTGCATTGGCCAAGCTTCACGAACTTGGATTCGCGCATGGAGATGTTAAATTGAACAACATGATGATCGATGTCCACGGCTTTGGGCATATGCTTGACATGGGCAGTGTGCGGCCTGTTGACAGCTGTGTAAGCGAGGAAGATAAATATTACCTGCGTTTGTGGGCTCCTGAACTTGCGAAATCACAGCACACGTCGCAAAAGACATGTCTGAAGCGTGGCGCCCTCGATGTGTGGGCCTTAGGGTTGGCAATCTTCGAGTTCGTCTGCTTCAACCGACTTCCTTACAGCCTTTCGAATCTGCCGAGTTCATTCTGGTCGAGAGTTGAACACCTTTCGCGCCTTCGCCTCTCAGATTTCTCTGTCAAGGATTGTAACGAATCTGATCCAGCAGTGATGGGAATTGTTGTTCAATTTCTAAATCCAGACCCTCAAGAGCGCCCTGAACTCCCGAAATTCGTCAACAGTTACACCTTCTTTCAGCAAGCCCCTGGAGTTACTTCTCATCTCACTAGGATTCCAACTACCGAACTTTCTTCACATCGGATGTAGGGATCCATCGATACTAGTAAGGATCTGCGATCGCTCCGGTGCCCGTCAGTGGGCAGAGCGCACATCGCCCACAGTCCCCGAGAAGTTGGGGGGAGGGGTCGGCAATTGAACGGGTGCCTAGAGAAGGTGGCGCGGGGTAAACTGGGAAAGTGATGTCGTGTACTGGCTCCGCCTTTTTCCCGAGGGTGGGGGAGAACCGTATATAAGTGCAGTAGTCGCCGTGAACGTTCTTTTTCGCAACGGGTTTGCCGCCAGAACACAGCTGAAG

**ROP16-ME49：**

CCATTGACGCAAATGGGCGGTAGGCGTGTACGGTGGGAGGTCTATATAAGCAGAGCTCGTTTAGTGAACCGTCAGATCGCCTGGAGACGCCATCCACGCTGTTTTGACCTCCATAGAAGACACCGACTCTACTAGAGGATCTATTTCCGGTGAATTCGCCACCATGCATCATCACCATCACCATAAAGTGACCACGAAAGGGCTTGCTTTTGCTCTTGCACTGTTGTTTTGTACACGCTGCGCAACTGCACGATACATGTCGTTTGAGGAAGCGCAAAAAGCAAGTGAAGCAGCGAAGCGCCAGATTGCCACACTCCCCTCTCCAGATTCTACTCTTTCGAATCCAGGTAGCAAGCATAGAAACCGGGGAGGGTCTCCTGCGGCAGGGCAACCTTCTCAATCCACACTACAACCTGAACAAGCGGCGGCTGAAGTAGGTCTCGGTGCTGGTGGCTCGACTCAGGGGCAGGGACGCACCGGTGGCAGCGCGGGTGCTAGAGAGGAGCGGAGGAGTCCTTCCCCCCAATCTGCTTATCCGGCGACTAGCTCAGCCTCGCTAAGGGGCTACCAAACCCAGCTTTCACCCTCGCATCTTCCACCACGCAGCAGCGGACCGGGAGGATGGTTTCCAACAGAGTCAATATTTACGCCATGGAGTTCTCCGCCGCAACCATTGACACAACGAAAGCCATCTCTATCTGGGGTGGTCGTTACCGAATTTCAAGAGCCACAAGAACAGTATGGCGCAGCGAGCAGTCTTGCGTCCTCGCCAAAGCGATACGTCAGTGGCGCAAGCTCGAGTGCATTGTCAGGAAAGGCGGTGCCAACGCCTGCGTCGCTTGGTCAAGAAAATCCTCTTTTCCCTGTTCAGAGCGCTACATTGGATTCAGGAATACAGTCTCCGGCACAAGAGCGTCGGGGATCCCCTCAAAGACAGATTGCGATGTCGACCGAAAATCCAGCGGATAGCGGCGCCTCGCAGCTTGCCTCCAGTGTTTCTAGTTATGTAGCAGTACAAACTCCTCATGTGAAACGTTCAGAACGCATCCGGCGCGTTCGACTTTCAGAAGAGGGTCTGGAAGAAGTTCAGCAGCTGAAAGCAGCTGCCGCACAGCTTCTCGTAGCGGTTCCGGACTATGAGGCAATGCGGGCTGTTCTGCAAGAGGCGGTCCTCTCAGAACAGAGGGTTGCTACCCGTAAGCGGAAGAGAAAGCAACCTCCAGGAGCGGTGGAGTCAGCTGTTGACGAAGTGTTCCCTCCAAATGAGCGTGTCATGATGATAAATGCCAACGGAGTGCCGATCGCTCTATACAATCGTGGGCACCTCGGCAGTGGACATTTCGGGGCTGTCATCAAGGCCAGCTTAGACGATGGGACGTTGTATGCAGCGAAGGTGCCGTACAGCCAGATCGTCCCGAATGCTGATGCCACGTCAGCAGAACTGGAGGCGGAAATTTCCTCAGCTAGGGCGGAGTTGGTAAAGACAATTCGACAGGAGTTGGATGTTCGGGATAAGCTGGTGGCTAAAGGGCTCACACTTACAGAGACTGCGGAGCAATACGGTCTACCATTGTGCCAAATGACTTTAACGCTTCCTGAGAACAAAGCAACCGTGGTACGTCGAGGTTCTCGACTCGTTGTCGTGTCTAAAGAAGTCATGCTGCTGCCATTAATTGATGGCTCCCCATCGAACAGTCTAGTCCAGTCGCAACCACCATTTCTCTTCCAGCGAGCTGTGGCAAGGGAAGCAATTATTGCATTGGCCAAGCTTCACGAACTTGGATTCGCGCATGGAGATGTTAAATTGAACAACATGATGATCGATGTCCACGGCTTTGGGCATATGCTTGACATGGGCAGTGTGCGGCCTGTTGACAGCTGTGTAAGCGAGGAAGATAAATATTACCTGCGTTTGTGGGCTCCTGAACTTGCGAAATCACAGCACACGTCGCAGCAGACATGTCTGAAGCGTGGCGCTCTCGATGTGTGGGCCTTAGGGTTGGCAATCTTCGAGTTCGTCTGCTTCAACCGACTTCCTTACAGCCTTTCGAATCTGCCGAGTTCACTCTGGTCGAGAGTTGAACACCTTTCGCGCCTTCGCCTCTCAGATTTCTCTGCCAAGGATTGTAACGAATCTGATCCAGCAGTGATGGGAATTGTTGCTCAATTTCTAAATCCAAATCCTGAAGAGCGCCCTGAACTCCCGAAATTCGTCAGCAGTTACACCTTCTTTCGGCAAGCCCCTGGAGTTACTTCTCATCTCACTAGGATTCCAACTACCGAACTTTCTTCACATCGGATGTAGGGATCCATCGATACTAGTAAGGATCTGCGATCGCTCCGGTGCCCGTCAGTGGGCAGAGCGCACATCGCCCACAGTCCCCGAGAAGTTGGGGGGAGGGGTCGGCAATTGAACGGGTGCCTAGAGAAGGTGGCGCGGGGTAAACTGGGAAAGTGATGTCGTGTACTGGCTCCGCCTTTTTCCCGAGGGTGGGGGAGAACCGTATATAAGTGCAGTAGTCGCCGTGAACGTTCTTTTTCGCAACGGGTTTGCCGCCAGAACACAGCTGAAGCTTCGAGGGGCTCGCATCTCTCCTTCACGCGCCCGCCGCCCTACC

**ROP16-VEG：**

ATGTCGTAACAACTCCGCCCCATTGACGCAAATGGGCGGTAGGCGTGTACGGTGGGAGGTCTATATAAGCAGAGCTCGTTTAGTGAACCGTCAGATCGCCTGGAGACGCCATCCACGCTGTTTTGACCTCCATAGAAGACACCGACTCTACTAGAGGATCTATTTCCGGTGAATTCGCCACCATGCATCATCACCATCACCATAAAGTGACCACGAAAGGGCTTGCTTTCGCTCTTGCACTGTTGTTTTGTACACGCTGCGCAACTGCACGATACATGTCGTTTGAGGAAGCGCAAAAAGCAAGTGAAGCAGCGAAGCGCCAGATTGCGACACTCCCCTCTCCAGATTCTCCTCTTTCGAATCCAGGTAGCAGGCATAGAAACCGGGGAGGGTCTCCTACGGCAGGGCAACCTTCTCAATCCACACTACAACCTGAACAAGCGGCGGCTGAAGTAGGTCTCGGTGCTGGTGGCTCGACTCAGGGGCAGGGACGCACCGGTGGCAGCGCGGGTGCTAGAGAGGAGCGGAGGAGTCCTTCCCCCCAATCTGCTTATCCGGCGACTAGCTCAGCCTCGCTAAGGGGCTACCAAACCCAGCTTTCACCCTCGCATCTTCCACCACACAGTAGCGGACCGGGAGGATGGTTTCCAACAGAGTCAATATATACGCTATGGAGTTCTCCGCCGCAACGATTGACACATCGAAAGCCATCTCTATCTGGGGTGGTCGTTACCGAATTTCAAGAGCCACAAGAACAGTATGGCGCAGCGAGCAGTCTTGCGTCCTCGCCAAAGGGATACGTCGGTGGCGCAAGCTCTAGTGCATTGTCAGGAAAGGCGGTGCCGACGCCTGCGTCGCTTGGTCAAGAAAATCCTCTTTTTCCTGGTCAGAGCGCTACATTGGATTCAGGAATACAGTCTCCGGCACAAAAGCGTCGGGGATCCCCTCAAAGACAGAGTGCGATGCCGACCGGAAATCCAGCAGATAGCGGCGCCTCGCAGCTTGCCTTCAGTCATTCTAGTTATGTATCAGTACAAGCTTCTCTTGCGAAACGTTCAGAACGCATCCGGCGCGTTCGACTTTCAGAAGAGGGTCTGGAAGAAGTTCAGCAGCTGAAAGCAGCTGCCGCACAGCTTCTCGTAGCGGTTCCGGACTATGAGGCAATGCGGGCTGTTCTGCAAGAGGCGGTCCTCTCAGAACAGAGGGTTGCTGCCCGTAAGCGGAAGAGAAAGCAACCTCCAGGAGCGGTGGAGTCAGCTGTTGACGAAGTGTTTCCTCCAAATGAGCGTGTCATGATGATAAATGCCAACGGAGTGCCGATCGCTCTATACAATCGTGGGCACCTCGGCAGTGGACATTTCGGGGCTGTCATCAAGGCCAGCTTAGACGATGGGACGCTGTATGCAGCGAAGGTGCCGTACAGCCAGATCGTCCCGAATGCTGATGCCACGTCAGCAGAACTGGAGGCGGGAATTTCCTCAGCTAGGGCGGAGTTGGTAAAGACAATTCGACAGGAGTTGGATGTTCGGGATAAGCTTGTGGCTAAAGGGCTCACACTTACAGAGACTGTGAGCCAATACGGTCTGCCATTGTGCCAAATGACTTTAACGCTTCCTGAGAACAAAGCAACCGTGGTACGTCGAGGTTCTCGACTCTTTGTCGTGTCCAAAGAAGTCATGCTGCTGCCATTAATTGATGGCTCCGCATTGAACAGTCTAGTCCAGTCGCAACCACCATTTCTCTTGCAGCGAGCTGTGGCAAGGGAAGCAATTCTTGCATTGGCCAAGCTTCACGAACTTGGATTCGCGCATGGAGATGTTAAATTGAACAACATGATGATCGATGTCCACGGCTTTGGGCATATGCTTGACATGGGCAGTGTGCGGCCTGTTGACAGCTGTGTAAGCGAGGAAGATAAATATTACCTGCGTTTGTGGGCTCCTGAACTTGCGAAATCACAGCACACGTCGCAAAAGACATGTCTGAAGCGTGGCGCCCTCGATGTGTGGGCCTTAGGGTTGGCAATCTTCGAGTTCGTCTGCTTCAACCGACTTCCTTACAGCCTTTCGAATCTGCCGAGTTCCTTCTGGTCGAGAGTTGAACACCTTTCGCGCCTTCGCCTCTCAGATTTCTCTGTCAAGGATTGTAACGAATCTGATCCAGCAGTGATGGGAATTGTTGCTCAATTTCTAAATCCAGACCCTCAAGAGCGCCCTGAACTCCCGAAATTCGTCAACAGTTACACCTTCTTTCAGCAAGCCCCTGGAGTTACTTCTCATCTCACTAGGATTCCAACTACCGAACTTTCTTCACATCGGATGTAGGGATCCATCGATACTAGTAAGGATCTGCGATCGCTCCGGTGCCCGTCAGTGGGCAGAGCGCACATCGCCCACAGTCCCCGAGAAGTTGGGGGGAGGGGTCGGCAATTGAACGGGTGCCTAGAGAAGGTGGCGCGGGGTAAACTGGGAAAGTGATGTCGTGTACTGGCTCCGCCTTTTTCCCGAGGGTGGGGGAGAACCGTATATAAGTGCAGTAGTCGCCGTGAACGTTCTTTTTCGCAACGGGTTTGCCGCCAGAACACAGCTGAAGCTTCGAGGGGCTCGCATCTCTCCTTCACGCGCCCGCCGCCCTACCTGAGGCCGCCATCCACGCCGGTTGAGTCGCGTTCTGCCGCCTCCCGCCTGTGGTGCCTCCTGAACTGCGTCCGCCGT

**Sequence Comparison Chart:**

**ROP16-RH：**


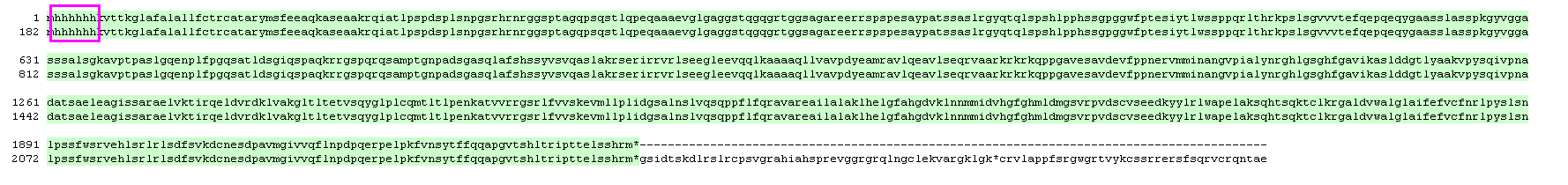


**ROP16-ME49：**


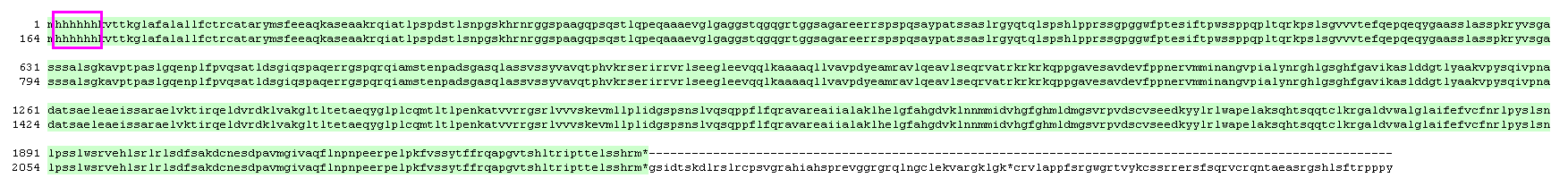


**ROP16-VEG：**


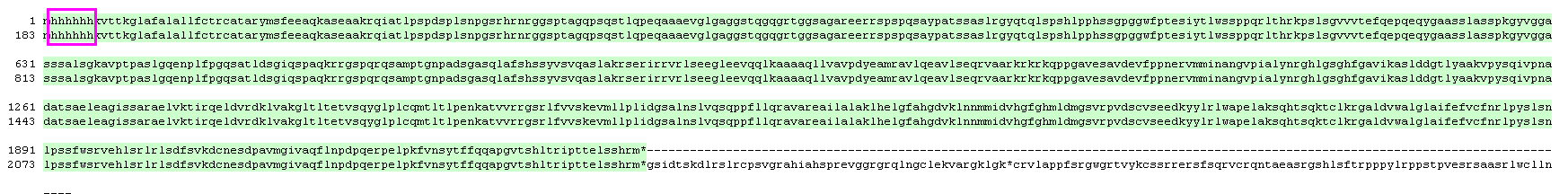


**Primer Sequences:**

| Gene names | Sequences |
| --- | --- |
| ROP16-RH-E/B-F | AGAGGATCTATTTCCGGTGAATTCGCCACCATGCATCATCACCATCACC |
| ROP16-RH-E/B-R | AGATCCTTACTAGTATCGATGGATCCCTACATCCGATGTGAAGAAAGTT |
| ROP16-ME49-E/B-F | AGAGGATCTATTTCCGGTGAATTCGCCACCATGCATCATCACCATCACC |
| ROP16-ME49-E/B-R | AGATCCTTACTAGTATCGATGGATCCCTACATCCGATGTGAAGAAAGTT |
| ROP16-VEG-E/B-F | AGAGGATCTATTTCCGGTGAATTCGCCACCATGCATCATCACCATCACC |
| ROP16-VEG-E/B-R | AGATCCTTACTAGTATCGATGGATCCCTACATCCGATGTGAAGAAAGTT |

**Mapping of plasmids:**

**ROP16-RH：**


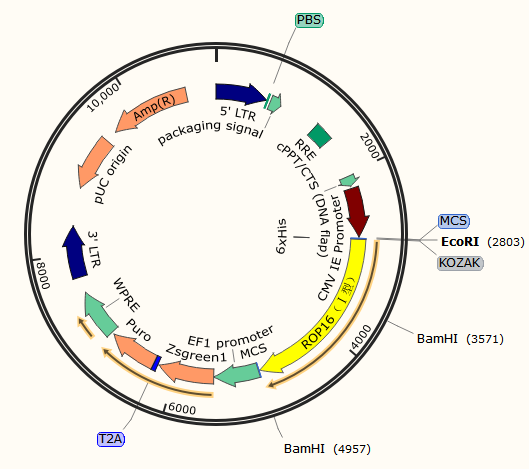


**ROP16-ME49：**


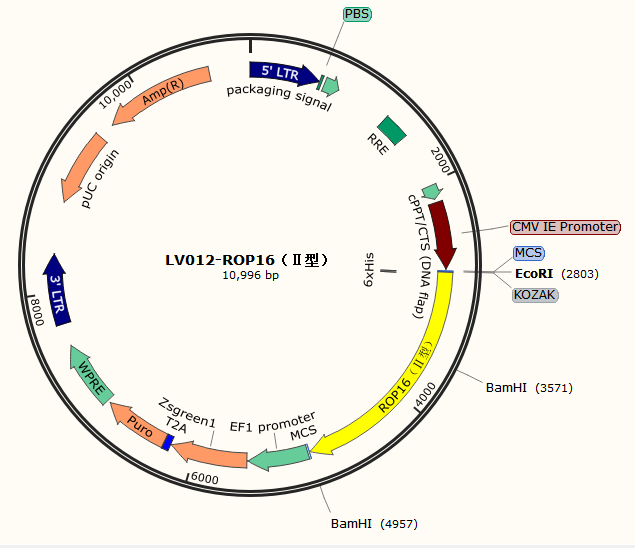


**ROP16-VEG：**


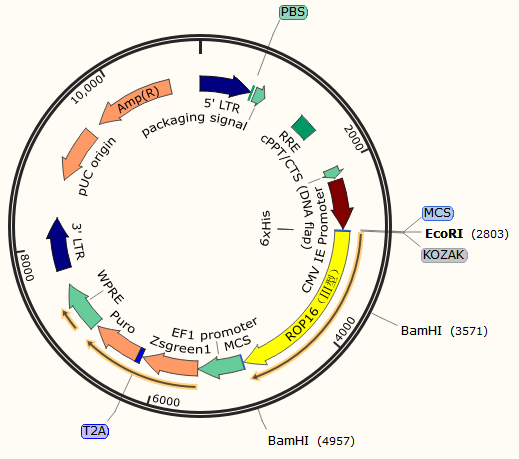

Supplement: Supplementary file 4 — Supplementary material 4. [file 13071_2025_6933_MOESM4_ESM.docx]
